# Vertical organization of microbial communities in Salineta hypersaline wetland, Spain

**DOI:** 10.3389/fmicb.2023.869907

**Published:** 2023-01-27

**Authors:** Zeina Bourhane, Christine Cagnon, Carmen Castañeda, Rafael Rodríguez-Ochoa, Jorge Álvaro-Fuentes, Cristiana Cravo-Laureau, Robert Duran

**Affiliations:** ^1^Université de Pau et des Pays de l’Adour, E2S UPPA, CNRS, IPREM, Pau, France; ^2^Estación Experimental de Aula Dei, EEAD-CSIC, Zaragoza, Spain; ^3^Departamento de Medio Ambiente y Ciencias del Suelo, Universidad de Lleida, Lleida, Spain

**Keywords:** ephemeral hypersaline lakes, hypersaline ecosystem, functional redundancy, reverse redox gradient, archaeal biomarkers

## Abstract

Microbial communities inhabiting hypersaline wetlands, well adapted to the environmental fluctuations due to flooding and desiccation events, play a key role in the biogeochemical cycles, ensuring ecosystem service. To better understand the ecosystem functioning, we studied soil microbial communities of Salineta wetland (NE Spain) in dry and wet seasons in three different landscape stations representing situations characteristic of ephemeral saline lakes: S1 soil usually submerged, S2 soil intermittently flooded, and S3 soil with halophytes. Microbial community composition was determined according to different redox layers by 16S rRNA gene barcoding. We observed reversed redox gradient, negative at the surface and positive in depth, which was identified by PERMANOVA as the main factor explaining microbial distribution. The Pseudomonadota, Gemmatimonadota, Bacteroidota, Desulfobacterota, and Halobacteriota phyla were dominant in all stations. Linear discriminant analysis effect size (LEfSe) revealed that the upper soil surface layer was characterized by the predominance of operational taxonomic units (OTUs) affiliated to strictly or facultative anaerobic halophilic bacteria and archaea while the subsurface soil layer was dominated by an OTU affiliated to *Roseibaca*, an aerobic alkali-tolerant bacterium. In addition, the potential functional capabilities, inferred by PICRUSt2 analysis, involved in carbon, nitrogen, and sulfur cycles were similar in all samples, irrespective of the redox stratification, suggesting functional redundancy. Our findings show microbial community changes according to water flooding conditions, which represent useful information for biomonitoring and management of these wetlands whose extreme aridity and salinity conditions are exposed to irreversible changes due to human activities.

## Introduction

Saline lakes are numerous and geographically widespread in inland ecosystems, particularly in arid and semi-arid areas (Williams, 1996, 2002). Endorheic basins cover approximately one-tenth of the Earth’s surface (Casamayor et al., 2013). They are rare in Europe (Casamayor et al., 2013), playing crucial ecosystem services. Particularly, as they are important habitats for migrating birds, they are included in biodiversity-protected areas under the RAMSAR convention (Ramsar Convention Secretariat, 2010). However, they are threatened by human activities and anthropogenic freshwater inputs (Williams, 2002). Saline lakes are very dynamic environments, with soil and water salinity and temperature fluctuations and thus highly responsive to climate changes (Menéndez-Serra et al., 2021). Among inland saline lakes, athalassohaline lakes, known for the lack of connections to marine environments, are characterized by a high content of sulfate, carbonate, and ionic composition different from that observed in seawater (Demergasso et al., 2008). The ecology of athalassohaline lakes is influenced by a high content of “chaotropic” ions such as Ca^2+^ and Mg^2+^ (Oren, 2015), having major effects on the abundance and growth of microbes (Gasol et al., 2004; Oren, 2013). Studies on microbial communities in several athalassohaline lakes revealed the presence of previously unidentified microorganisms, adapted to salinity fluctuations (Jiang et al., 2006; Demergasso et al., 2008; Menéndez-Serra et al., 2021). As athalassohaline lakes vary in ionic composition according to their location, they exhibit distinct microbial communities more divergent than that observed for thalassohaline environments (Pagaling et al., 2009; McGenity and Oren, 2012), representing genomic islands with limited microbial exchange and own evolution (Pagaling et al., 2009). Nevertheless, athalassohaline ecosystems represent specific habitats for halophilic prokaryotes and eukaryotes, most of them being also polyextremophile able to survive under extreme conditions of salinity, pH, and UV radiation (McGenity and Oren, 2012). The halophilic microorganisms include various aerobes and anaerobes covering diverse functional groups, such as heterotrophs, oxygenic and anoxygenic phototrophs, lithotrophs, methanogens, and methylotrophs, fermenters, sulfate reducers, and sulfide oxidizers (Sorokin et al., 2011; Lanzén et al., 2013).

The Salineta at “Saladas de Bujaraloz-Sástago” (Spain), located in the south of Monegros, one of the most arid areas in Europe, is a lake of an endorheic complex constituted by a large set of inland ephemeral saline lakes subjected to temporary flooding according to seasons (Castañeda and Herrero, 2005; Casamayor et al., 2013; Menéndez-Serra et al., 2021). Similar microbial communities have been described among different saline lakes, constituted by microorganisms with a competitive advantage, characteristic of these dynamic ephemeral lakes (Casamayor et al., 2013; Menéndez-Serra et al., 2019, 2021). The saline lakes are vulnerable to diffuse pollution from agricultural activities surrounding fields and pig farming (Castañeda et al., 2015). The transfer of pollutants is enhanced by the loamy-sandy soil texture and the abundance of gypsum (Castañeda et al., 2015). Chronic accumulation of pollutants is known to contribute to the disruption of microbial community diversity and biogeochemical cycle functions (Bordenave et al., 2004; Duran and Cravo-Laureau, 2016). It is thus of paramount importance to obtain a characterization of the Salineta wetland prior to the intensification of the farming activities that will serve as a baseline for the management of this RAMSAR-protected area.

Among these saline lakes, the Salineta wetland, surrounded by newly irrigated areas, is characterized by three different landscapes corresponding to water flooding conditions: usually submerged soil, intermittently flooded soil, and soil being vegetated with halophytes. The Salineta hydric regime, depending on the rains and the shallow water table dynamics (Castañeda and García-Vera, 2008), contributes to the development of contrasting soil surface microenvironments (Dominguez-Beisiegel et al., 2011), leading to distinct soil redox conditions. We hypothesized that different microbial communities inhabit these three different water-related soil environments corresponding to microbial communities adapted to the different soil and water characteristics of ephemeral saline lakes. To test our hypothesis, we characterized the microbial community composition and inferred the metabolic functional profiles involved in carbon, nitrogen, and sulfur cycles in the wetland soil at different depths according to the soil layering observed in both dry (summer) and wet (winter) periods.

## Materials and methods

### Site description

**FIGURE 1:**
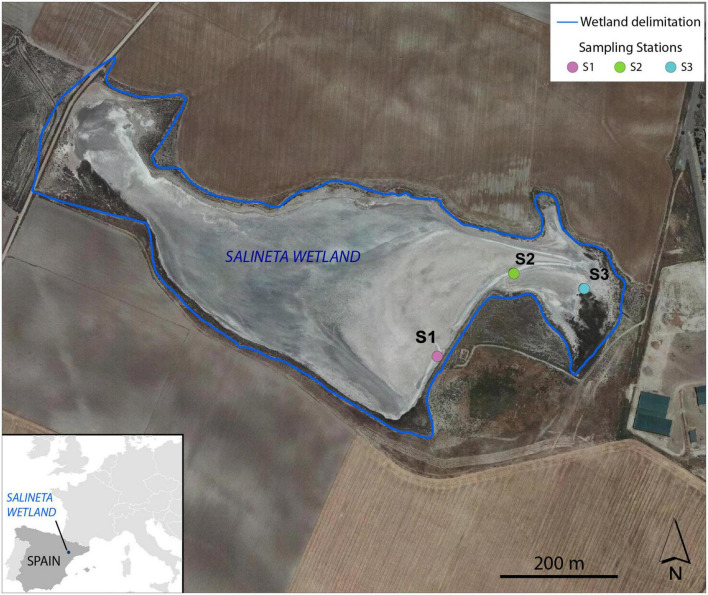
Location of the sampling stations (S1, S2, and S3) at Salineta wetland in the orthophotograph of PNOA 2015 (National Geographic Institute of Spain).

The Salineta at “Saladas de Bujaraloz-Sástago” (NE, Spain) is located in Monegros, one of the most arid regions in Europe, with a mean annual precipitation of 350 mm and an annual reference evapotranspiration (ET_0_) of 1,225 mm (Castañeda and Herrero, 2005). It is included in the RAMSAR convention list for bird protection and in the Natura, 2000 network. The Monegros endorheic complex contains more than a hundred seasonal inland saline lakes whose salinity is linked to climatic aridity and geologic materials. Salineta, with 23 ha, is one of the northernmost saline lakes of the complex and has the greatest presence of water (Castañeda and García-Vera, 2008; Figure 1). The groundwater discharges at the lakebed coming from two long-standing saline aquifers (salinity 294 ± 34 g/l) whose high salinity is due to the chemical and lithological characteristics of the sediments. Wetland soils are very saline and rich in gypsum and carbonates (Menéndez-Serra et al., 2019).

### Sampling

Three different soil stations were selected in the Salineta wetland based on the habitat maps (Conesa et al., 2011). S1, S2, and S3 corresponded to usually submerged soil, intermittently flooded soil, and soil covered with halophytes (Figure 1). These stations were considered representative of water-related soil conditions in these ephemeral saline lakes. In each sampling station, triplicate cores of soil surface horizons up to 30–40 cm were sampled by an auger (7 cm in diameter). For each site, soil layers were identified according to their color following the Munsell color chart, which relies on different soil chemical and physical characteristics (Kalev and Toor, 2018). Soil samples for molecular and chemical analysis were taken within the three soil layers. Two sampling campaigns, in winter (March 2019, wet period) and summer (September 2019, dry period), were performed. For station S1, three different soil layers were identified by their color (L1, greenish black 0–5 cm; L2, olive gray 5–18 cm; L3, yellowish brown 18–25 cm), while only two layers (L1 and L2) for stations S2 and S3 were distinguished (Supplementary Figure 1). Similar vertical layering was collected in both seasons. Thus, 42 samples ([3 stations × layers (3 for S1, 2 for S2 and S3) × 2 seasons] × 3 replicates) were obtained. For each layer, soil samples were collected with a sterile spatula. After homogenization, the soil samples were distributed in 2 ml sterile cryotubes frozen in liquid nitrogen and were conserved at −80°C for microbial analysis. For physical–chemical analyses, samples were collected in 25-ml tubes and stored at −20°C until use.

### Physical–Chemical analysis

Gas emissions (CO_2_, N_2_O, and CH_4_) were measured *in situ* using closed chambers placed on top of the soil and gas chromatography (Agilent 7890B) equipped with a flame ionization detector (FID) coupled to a methanizer for CO_2_ determination (Franco-Luesma et al., 2020). For station S3, in order to limit the effect of plants, the chambers were placed in a part of the soil surrounded by halophytes but without plants inside.

Physical–chemical parameters [temperature, pH, salinity, electrical conductivity (EC), redox potential (ORP), and dissolved oxygen] were determined in the field with a multiparameter system (WTW Multi-197i) (Ben Salem et al., 2016). Calcium carbonate equivalent (CCE), gypsum content, organic matter (OM), particle-size distribution, and major ions (ammonium, nitrite, nitrate, fluoride, chloride, bromide, sulfate, bicarbonate, sodium, potassium, calcium, and magnesium) were determined (Castañeda et al., 2017). Briefly, the Bernard calcimeter method (reaction with HCl) was used to determine the CCE, gypsum content was determined by thermogravimetry, and OM by spectrophotometry after chromic acid digestion using a UV/V UNICAM 8625 spectrophotometer. Particle-size distribution was assessed by laser diffraction (Malvern MASTERSIZER 2000). Ion chromatography Metrohm 861 (Metrohm AG, Herisau, Suisse) was used to determine the major ions.

### DNA extraction, PCR amplification, and sequencing

Microbial communities were extracted by suspending 250 mg of ground soil (in liquid nitrogen) in 1.2 ml of distilled water. After mixing for 45 s at 6,000 rpm using Precellys Evolution homogenizer (Bertin technology, Montigny-le-Bretonneux, France) and centrifugation for 30 s at 10,000 × *g* the supernatant was recovered. This operation was performed six times. A total of 7.2 ml for each sample was filtered using 0.2-μm MicroFunnel (PALL Corporation, Portsmouth, United Kingdom). DNA was extracted from the cut filters using PowerSoil DNA Kit (MoBio Laboratories Inc., Courtaboeuf, France) following the manufacturer’s instructions with few modifications (Giloteaux et al., 2013). The region V4–V5 of 16S rRNA was amplified using prokaryotic universal primers U515-532-GTGYCAGCMGCCGCGGTA and U909-928-CCCCGYCAATTCMTTT (Wang and Qian, 2009), which match 80% of the Bacteria domain and the same for Archaea domain (tested with).^1^ The primers include the linkers for barcoding provided by the GenoToul platform. The amplifications were performed in triplicates (Bourhane et al., 2022). Briefly, the amplifications were performed in a total volume of 25 μl, using 12.5 μl of AmpliTaq Gold 360 Master Mix, 0.5 μl of each forward and reverse primers (20 μM), and 5 μl of the DNA extract, as follows: 95°C for 10 min, 30 cycles of 95°C for 30 s, 60°C for 30 s, 72°C for 40 s, and a final extension step at 72°C for 10 min. For each sample, the three PCR products were pooled and sequenced by Illumina-MiSeq (paired-end 2 × 250 bp) at the GenoToul platform (Toulouse, France). The complete protocol is available at https://sites.google.com/site/olivierzembwebsite/16s-sequencing.

### Sequence data analysis

Sequence data analysis was performed (Lanzén et al., 2021). Briefly, the sequences were trimmed as follows: reads were overlapped using *vsearch* version 2.7.1 (Rognes et al., 2016), then primers were removed using *cutadapt* version 1.15 (Martin, 2011). The remaining sequences were de-replicated and sorted by abundance using *vsearch*. Then, they were used to cluster the reads into operational taxonomic units (OTUs) with a minimum linkage of one nucleotide using SWARM version 2.2.1 (Mahé et al., 2015). Abundances of unique sequences across samples were retained and used to build an OTU contingency table based on SWARM version 2.2.1 output using SLIM (Dufresne et al., 2019). Singleton OTUs were discarded and then applied reference-based and *de novo* UCHIME chimera filtering as implemented in *vsearch* (Lanzén et al., 2012), with SilvaMod version 138 as the reference database (Lanzén et al., 2021).^2^ Then, we applied LULU post-clustering curation with a minimum 97% similarity cutoff point (Frøslev et al., 2017) to correct the remaining sequencing artifacts and merge OTUs with intra-specific or intra-genomic differences. Final SWARM OTUs were aligned to SilvaMod version 138 using *blastn* version 2.6.0 + and taxonomically classified using CREST version 3.1.0 (Lanzén et al., 2021). The data set was deposited in the NCBI Sequence Read Archive (SRA) database under accession number ID PRJNA763109.

Predicted cross-contaminant reads were removed using a procedure analogous to UNCROSS (Edgar, 2016) by setting sample-specific OTU abundances to zero when encountered at an abundance below 2% of the average OTU abundance across samples. In addition, in order to compensate for the possible bias introduced by uneven sequencing depths, OTUs present with a maximum abundance across samples below 0.01% were discarded (Elbrecht et al., 2018).

The functional predictions of microbial communities involved in the main biogeochemical cycles were determined using Phylogenetic Investigation of Communities by Reconstruction of Unobserved States, version 2 (PICRUSt2) from the 16S rRNA gene sequences (Douglas et al., 2020). The recommended maximum NSTI cutoff point of 2 was implemented by default in PICRUSt2 to prevent unconsidered interpretation of overly speculative inferences (Douglas et al., 2020), which excluded 2.2% of OTUs (211 out of 9,557). These removed OTUs represented 1.1% of the relative abundance of the microbial community. The Kyoto Encyclopedia of Genes and Genomes (KEGG) databases were used for functional prediction annotation and metabolic pathways analysis. The weighted nearest sequenced taxon index (NSTI) was calculated to assess the accuracy of PICRUSt analysis (Langille et al., 2013). The KOs associated with nitrogen, sulfur, and carbon metabolisms were identified.

### Statistical analysis

Statistical analyses were run in R environments^3^ using vegan packages (Oksanen et al., 2019). Alpha diversity indices (including species richness, Shannon—Wiener, and evenness) of microbial community were calculated for each sample based on the rarefied OTU table using the alpha function of the vegan package in R.

Multiple comparison of gas flux was computed by applying the least significant difference (LSD) test within the “agricolae” package using the “LSD.test” function for multiple comparisons after the ANOVA test. Permutational multivariate analysis of variance (Permanova) was performed to estimate the effect of physical–chemical parameters on the microbial communities (999 permutations). Microbial community similarity was performed by non-metric multidimensional scaling (NMDS), based on Bray–Curtis index, correlating communities with physical–chemical parameters. The significant environmental variables were fitted to the NMDS as vectors with the envfit function on the R statistics software.

Linear discriminant analysis effect size (LEfSe) (Segata et al., 2011) was performed to determine significant seasonal and spatiotemporal OTUs biomarkers among the most abundant genera. Briefly, the non-parametric Kruskal–Wallis (KW) sum-rank test was first applied to detect significant differential taxa abundance (*p* < 0.05). Then, the biological consistency was investigated by performing a Wilcoxon pairwise test (*p* < 0.05). The linear discriminant analysis (LDA) threshold was set up to 2 and 1,000 bootstrap interactions. The chordogram profile, performed using the chordDiagram function, showed the distribution of functions in each sample. Ternary plot performed using the PAST software presented the distribution of functions according to the layer depths. Principal component analysis (PCA) showed the distribution of samples and the functional genes correlating microbial community with the environmental factors.

## Results

### Physical–Chemical characterization

Microbial activities were estimated by the production of CO_2_, N_2_O, and CH_4_ in soil from each station (Supplementary Table 1). The main difference was observed for CO_2_ fluxes, with station S3 showing higher CO_2_ fluxes (ANOVA, *p* < 0.01) in both seasons. The three stations showed similar CH_4_ fluxes in summer, but they exhibited different CH_4_ fluxes in winter (ANOVA, *p* < 0.1) (Supplementary Table 1). Station S3 differs from stations S1 and S2 in physical–chemical parameters (Supplementary Table 2 and Supplementary Figure 2). Such a difference was expected since the sampling stations represent a gradient of surface water persistence in the wetland. The soil is usually submerged at S1, intermittently flooded at S2, and covered by halophytes at S3. Furthermore, considering all physical–chemical parameters, significantly different characteristics were also observed according to layers (two-way ANOVA, *p* < 0.005), except at S1 with layer L3 having similar physical–chemical characteristics as layer L2 (Supplementary Table 2). The NMDS showed that layer L1 is linked with higher ion concentrations and low (ORP, −155 ± 193 mV), while L2 has higher redox (ORP, 88 ± 118 mV) and soil texture (silt, sand, clay; Supplementary Figure 2). The differences between L1 and L2 were further supported by two-way ANOVA (Supplementary Table 2). Noteworthy, the layers showed distinct ORP with seasonal variability (Supplementary Table 2), probably related to the difference in soil temperature observed between winter (9°C) and summer (28°C). However, despite the observed differences, the general trend was that the negative ORP values of the soil surface (L1) shifted to positive ORP values in depth (L2 first and then L3).

### Microbial community composition

**FIGURE 2:**
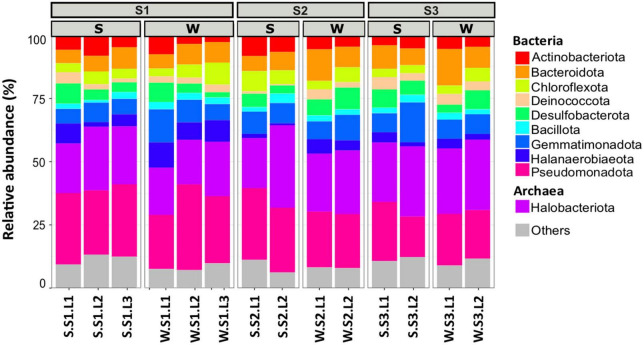
Microbial community composition of the 10 most abundant phyla in different layers (L1, L2, L3 or L1, L2) from each station (S1, S2, S3) at summer (S, dry period) and winter (W, wet period). Each bar plots represents the mean of three biological replicates (except for S.S1.L2, *n* = 2). Others represent phyla with relative abundance <5%.

To determine the microbial community organization, the microbial composition was characterized by 16S rRNA gene barcoding. A total of 483,501 reads were obtained from 42 samples. The rarefaction curves showed a plateau (Supplementary Figure 3), indicating that the sequencing effort was sufficient to assess the prokaryotic diversity, except for the sample S.S1.L2.R3 that has been removed for the analysis because the number of sequences obtained is low. After trimming, the retained 287,781 sequences were distributed within 1,158 OTUs (Table 1). The microbial community was composed of 74% of Bacteria (21 main phyla) and 26% of Archaea (5 main phyla) (Figure 2). The microbial richness and diversity indices were significantly different between the samples (ANOVA, *p* < 0.05, Table 1), showing that the microbial communities were affected by the conditions prevailing in the stations according to layers and seasons. In all stations, Pseudomonadota (15–33%), Gemmatimonadota (6–16%), Bacteroidota (6–13%), and Desulfobacterota (3–9%) phyla dominated the Bacteria, while Halobacteriota (18–31%) phylum dominated the Archaea (Figure 2), representing 93% of the Archaea. Although the samples showed similar patterns, it is worth noting that some differences can be observed. Especially, Bacteroidota were more abundant in layer L1 of stations S2 and S3 in winter, while Halobacteriota were more abundant in layer L2 of stations S2 and S3 in summer (Figure 2). In addition, the relative abundance of Actinobacteriota and Deinococcota decreased with depth and season. To further characterize the differences, multivariate analyses were conducted.

**TABLE 1:** Alpha diversity indices of prokaryotic community from the study sites located at the stations S1, S2, and S3 in Salineta wetland in winter (W) and summer (S) in different layers (L1, L2, and L3).

Indices		Stations						
		S1			S2		S3	
		L1	L2	L3	L1	L2	L1	L2
Reads	W	39126 ± 6540^a^	40536 ± 11948^a^	43489 ± 9118^a^	45297 ± 18095^a^	31602 ± 5187^a^	39515 ± 2538^a^	36334 ± 8543^a^
	S	34672 ± 3461^a^	25392 ± 19798^a^	19286 ± 2748^a^	29249 ± 5697^a^	28812 ± 8666^a^	38386 ± 8977^a^	31805 ± 10261^a^
Trimmed sequences	W	211867 ± 687^a^	26174 ± 6874^a^	27235 ± 4398^a^	22464 ± 2365^a^	20519 ± 3214^a^	22401 ± 2295^a^	22319 ± 4863^a^
	S	21206 ± 3915^a^	22380 ± 275^a^	12253 ± 1691^a^	17889 ± 2204^a^	17801 ± 5638^a^	22831 ± 6381^a^	19911 ± 6611^a^
Species richness (R)	W	1635 ± 58^ab^	1004 ± 229^c^	1106 ± 59^c^	1919 ± 69^a^	1337 ± 158^bc^	1878 ± 71^a^	1308 ± 323^bc^
	S	1253 ± 186^ab^	1032 ± 98^abc^	579 ± 44^b^	855 ± 232^b^	664 ± 135^b^	1706 ± 283^a^	848 ± 240^b^
Simpson *(1-D)*	W	0.98 ± 0.004^abc^	0.97 ± 0.002^c^	0.98 ± 0.004^bc^	0.98 ± 0.002^a^	0.95 ± 0.057^abc^	0.99 ± 0.003^ab^	0.98 ± 0.014^ab^
	S	0.98 ± 0.004^a^	0.97 ± 0.002^a^	0.98 ± 0.0005^a^	0.99 ± 0.0009^a^	0.99 ± 0.002^a^	0.99 ± 0.0008^a^	0.99 ± 0.001^a^
Shannon *(H)*	W	5.39 ± 0.11^bc^	4.77 ± 0.38^d^	4.96 ± 0.01^cd^	6 ± 0.09^a^	5.68 ± 0.19^ab^	5.91 ± 0.11^ab^	5.81 ± 0.06^ab^
	S	5.24 ± 0.34^ab^	4.98 ± 0.09^ab^	5.25 ± 0.23^ab^	5.21 ± 0.29^ab^	4.63 ± 0.61^b^	5.71 ± 0.06^a^	5.14 ± 0.27^ab^
Pielou Evenness *(E)*	W	0.73 ± 0.011^bc^	0.7 ± 0.03^c^	0.7 ± 0.0069^c^	0.8 ± 0.01^a^	0.8 ± 0.01^ab^	0.78 ± 0.01^ab^	0.81 ± 0.03^a^
	S	0.73 ± 0.032^a^	0.72 ± 0.003^a^	0.82 ± 0.03^a^	0.77 ± 0.02^a^	0.71 ± 0.11^a^	0.77 ± 0.02^a^	0.77 ± 0.02^a^

Mean ± SD are presented (*n* = 3; except for S.S1.L2, *n* = 2).

The same small letter indicates no significant difference of means compared by ANOVA, *p* < 0.05.

### Spatiotemporal microbial community distribution

**FIGURE 3:**
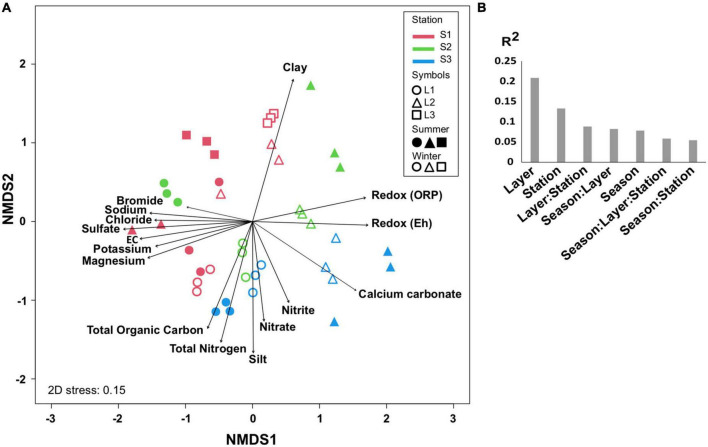
Comparison of microbial communities. **(A)** Non-metric multidimensional scale (NMDS) at OTU level in different layers (L1, L2, L3 or L1, L2) from each station (S1, S2, S3) at summer (S, dry period) and winter (W, wet period). The significant environmental variables are represented by vectors. **(B)** PERMANOVA partitioning (*R*^2^ value, *p* < 0.001) according to layers, stations, seasons, and their interactions. Three biological replicates (except for S.S1.L2, *n* = 2) were analyzed per sample.

The comparison of microbial communities by NMDS separated the layers L1, L2, and L3 in clusters and then according to the stations (S1, S2, and S3) and seasons (summer and winter) (Figure 3A), which was consistent with PERMANOVA indicating that the variation between samples was explained mainly by layers (*R*^2^ = 0.2, *p* < 0.001), stations, and seasons (Figure 3B). It is likely that the microbial community organization was driven by environmental factors, with L1 being controlled by ion concentrations, OM, and total nitrogen, whereas L2 was mainly influenced by redox (Figure 3A).

**FIGURE 4:**
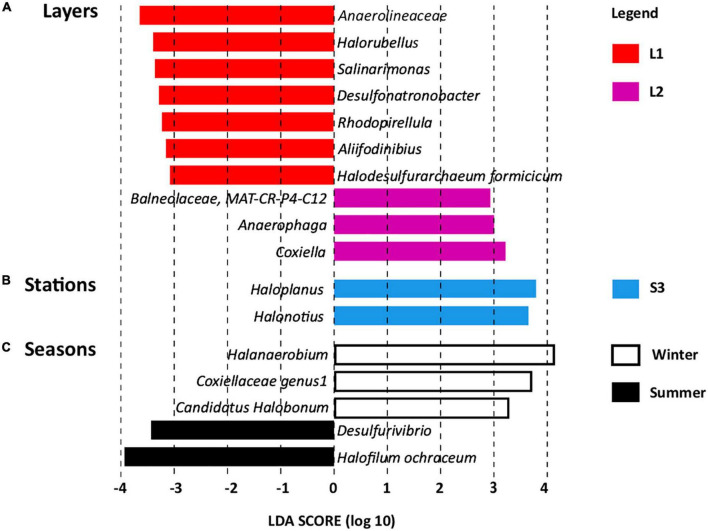
Linear discriminant analysis effect size (LEfSe) identifying microbial genera specifically more abundant according to **(A)** layers (L1 and L2), **(B)** stations (S1, S2, and S3), and **(C)** seasons (summer and winter, dry and wet periods, respectively).

Linear discriminant analysis effect size revealed OTUs found significantly more abundant according to layers (Figure 4A), stations (Figure 4B), and seasons (Figure 4C), which serve as biomarkers (Mehrshad et al., 2013). Layer L1 was characterized by seven OTU biomarkers, which are usually detected in hypersaline ecosystems, some of them being detected in salt-saturated ecosystems with pH around 8, conditions prevailing at Salineta wetland. These OTU biomarkers include five bacterial OTUs affiliated to the Anaerolineaceae family and the *Salinarimonas*, *Rhodopirellula*, *Desulfonatronobacter*, and *Aliifodinibius* genera; and two archaeal OTUs affiliated to the *Halorubellus* genus and *Halodesulfurarchaeum formicicum* (Figure 4A). Three OTUs related to *Coxiella* and *Anaerophaga* genera and the Balneolaceae family were the biomarkers for layer L2.

Regarding station biomarkers, only two OTUs were identified for station S3 that were affiliated with the archaeal *Haloplanus* and *Halonotius* genera (Figure 4B). These biomarkers further support the specificity of station S3 that differs from stations S1 and S2 in physical–chemical parameters (Supplementary Table 2 and Supplementary Figure 2), being covered by halophytes. Seasonal biomarkers were also identified (Figure 4C), three for winter (*Halanaerobium*, Coxiellaceae genus 1, and *Candidatus Halobonum*) and two for summer (*Halofilum ochraceum* and *Desulfovibrio*), supporting seasonal fluctuation, probably related to the salinity variations according to wet and dry periods.

### Functional distribution

**FIGURE 5:**
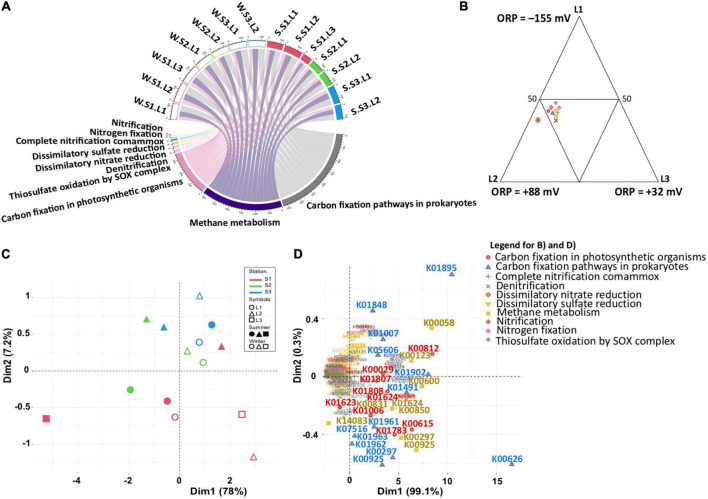
Predicted functional analysis for sulfur, nitrogen, and carbon metabolism inferred from 16S rRNA gene data (PICRUSt2). **(A)** Chordogram showing the distribution of functions among samples. **(B)** Ternary plot showing the distribution of functions among layers. **(C,D)** Principal component analysis (PCA) showing the distribution of samples based on the PICRUSt2 predictive functions, and the functional genes, respectively. Winter, W (wet period); Summer, S (dry period); Stations: S1, S2, S3; Layers: L1, L2, L3.

To estimate whether the functional capabilities involved in the main biogeochemical cycles (nitrogen, sulfur, and carbon) are affected by taxonomic composition variability, functional profiles were determined by PICRUSt2, with an NSTI score of 0.32 showing the accuracy of the prediction analysis. Although predictive, such an approach is a useful tool to obtain an overview of specific microbial process dynamics (Menéndez-Serra et al., 2019). Focusing on nitrogen, sulfur, and carbon metabolisms, the functional profiles were similar in all samples (Figure 5A and Supplementary Table 3). Considering the differences observed in the microbial community (Figure 3A), this observation indicated that the same metabolic functions are ensured by different microbial communities. Except for nitrification, the metabolic functions were equally distributed according to layers (Figure 5B), characterized by different redox (ANOVA, *p* < 0.05) suggesting functional redundancy. However, the PCA based on functional groups (Figure 5C) showed a dispersion of the microbial communities, confirming the distribution and differences observed with the phylogenetic analysis (Figures 3A, B). The distribution is explained by the presence of different KOs for each functional group (Figure 5D), confirming the functional redundancy.

### Metabolic function associations

**FIGURE 6:**
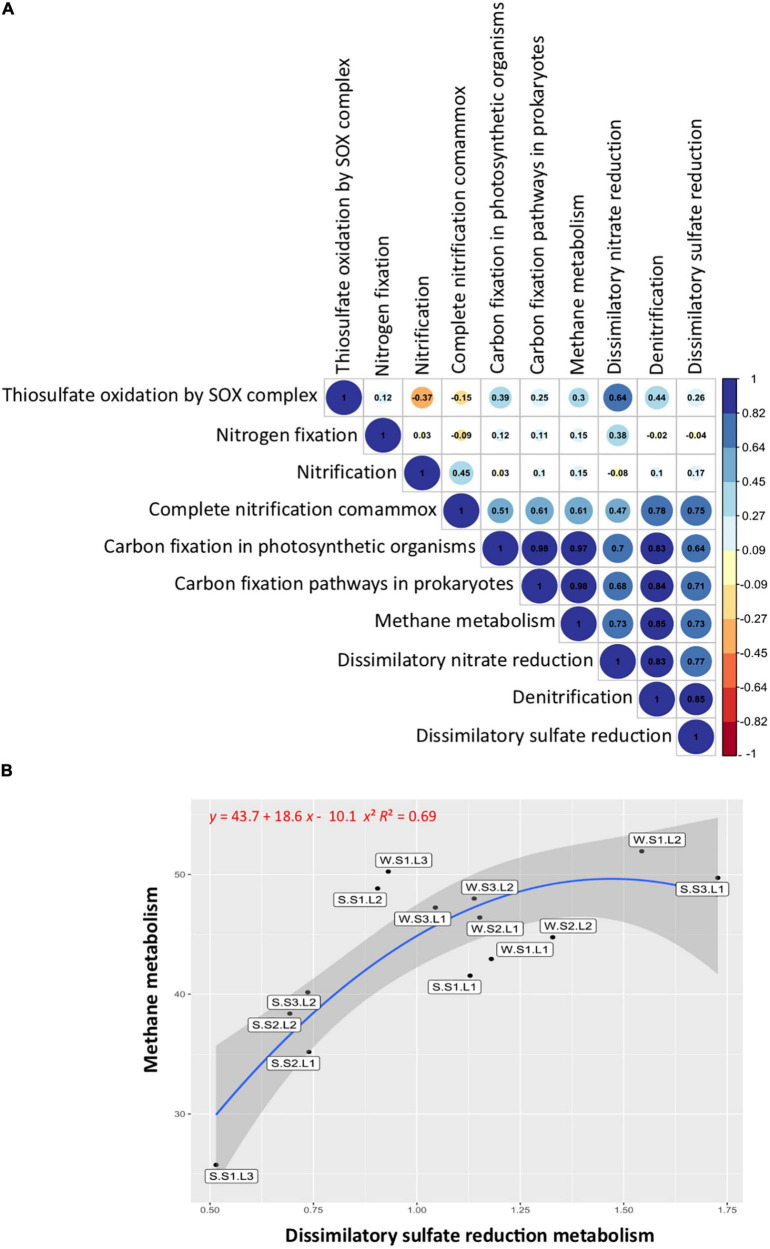
Functional group correlations and distributions. **(A)** Pearson’s correlations between sulfur, nitrogen, and carbon metabolisms. Blue, positive correlations; red, negative correlations. **(B)** Correlation between methane and dissimilatory sulfate reduction metabolisms. Labels indicate sample names: seasons, summer (S, dry period) and winter (W, wet period); stations (S1, S2, S3); layers (L1, L2, L3).

The correlation analysis between the studied functional groups (Figure 6A) showed a significant (*p* < 0.05) and positive correlation between the functions involved in the sulfur cycle, with the functions related to the nitrogen cycle including denitrification, dissimilatory nitrate reduction to ammonium (DNRA), and complete ammonium oxidation (COMAMMOX), but not significantly correlated with nitrogen fixation and nitrification. Noteworthy, the functions involved in the sulfur cycle were positively correlated with the functions of the carbon cycle (Figure 6A). When the methane metabolism functions (methanogenesis and methanotrophy) were abundant, the sulfate reduction functions were also abundant. Particularly, the layers showing the lowest redox at the S1 station exhibited abundant methane metabolism and sulfate reduction functions, especially layer L1 at both seasons (Figure 6B). In addition, it is important to note that these functions were more abundant during the wet season (winter), showing the effect of flooding on microbial functions.

## Discussion

The ephemeral saline lake of Salineta wetland is driven by the hydric regime (Castañeda and García-Vera, 2008) that results in three different landscapes (Dominguez-Beisiegel et al., 2011) observable according to the water flooding conditions: usually submerged soil (station S1), intermittently flooded soil (station S2), and soil being vegetated with halophytes (station S3). The microbial activities, estimated by gas fluxes (CO_2_, N_2_O, and CH_4_), were different in the three stations, being particularly observable in winter for methane fluxes. It has been shown that the production of greenhouse gases is affected by flooding and variations in salinity (Ringeval et al., 2010; Kroeger et al., 2017), which relies on the control of microbial activities by the dynamics of redox gradients (Reddy and DeLaune, 2008), suggesting that the flooding conditions affect the microbial activities in Salineta wetland. Consistently, we observed physical–chemical differences between the three landscapes, particularly according to ORP, but the more stricky observation was the stratification of the soil exhibiting significantly different physical–chemical characteristics according to layers. Especially, ORP negative values were observed at the soil surface shifting to positive ORP values with depth, despite the seasonal variations. Such observation constitutes a distinctive feature of the Salineta wetland in comparison to other subaqueous ecosystems where the ORP decreases with depth, such as in marine sediments (Kristensen, 2000). The seasonal presence of different types of salt crusts, the physical and chemical characteristics of soil and water, and the hydric regime of the Salineta wetland (Castañeda and García-Vera, 2008) as well as the OM content (Schultz, 2000) may explain the observed reversed redox gradient in the soil profile, which affects the microbial community assemblages. Future studies will benefit from the fine-scale determination of oxygen and sulfide micro-profiles to better characterize redox gradients in the soil.

The microbial community characterization by 16S rRNA gene meta-barcoding analysis revealed that above 26% of the sequences belonged to Archaea. Although the inherent biases of the sequencing approach for the quantification, it is important to note that Archaea have been found in similar proportion in hypersaline ecosystems, such as the Karak Salt Mine, Pakistan (Cycil et al., 2020), and ocean waters (Karner et al., 2001). Archaea often make the main component of the microbial community in hypersaline systems (Andrei et al., 2012). They have also been found abundant in many extreme ecosystems (Kan et al., 2006; Bruneel et al., 2008) and hydrocarbon-polluted sediments (Stauffert et al., 2014) where they play an important role in biogeochemical cycles influencing the emission of greenhouse gases (Offre et al., 2013). It has been demonstrated that the Archaea proportion reflects the influence of the differences in environmental parameters (Wang et al., 2020). The alpha diversity indices were significantly different between the samples (ANOVA, *p* < 0.05, Table 1), indicating that the microbial communities were affected by the conditions prevailing in the stations according to layers and seasons. The dominant Bacteria (Pseudomonadota, Gemmatimonadota, Bacteroidota, and Desulfobacterota) and Archaea (Halobacteriota) phyla observed in Salineta wetland include genera usually found dominant in sediment from hypersaline and saline lakes (Dong et al., 2006; Menéndez-Serra et al., 2019), microbial mats (Kirk Harris et al., 2013; Bolhuis et al., 2014; Mazière et al., 2021), and salt mines (Cycil et al., 2020).

The Pseudomonadota phylum was dominated by *Thiohalorhabdus* (4%), Acidithiobacillaceae group RCP1-48 (4%), and *Wenzhouxiangella* (2%) genera (representing, respectively, 8, 8, and 4% of the phylum) that have been found in hypersaline ecosystems (Sorokin et al., 2008; Caton and Schneegurt, 2012; Zhang et al., 2020). Members of the *Thiohalorhabdus* and *Acidithiobacillaceae* group RCP1-48 are known to play an important role in sulfur and iron compounds oxidation (Sorokin et al., 2008; Arce-Rodríguez et al., 2020), while *Wenzhouxiangella*, described as a predator of Gram-positive bacteria cells, exhibit proteolytic activity (Sorokin et al., 2020). The aerobic anoxygenic phototroph *Gemmatimonas* genus dominating the Gemmatimonadota (0.01, 0.1% of the phylum) has been detected in gypsum-rich soil (Yuan et al., 2021). Its ability to grow under micro-oxic conditions (Zeng et al., 2015) provides advantages to survive in intermittently flooded soil (Yuan et al., 2021), such as in the Salineta wetland. In addition, members of the *Gemmatimonas* genus have been found in the rhizosphere of alkali vegetation (Yue et al., 2020; Borsodi et al., 2021) playing a key role in vegetated saline soil, as OM-decomposing and polyphosphate-accumulating bacterium (Mau et al., 2015). The Bacteroidota was dominated by the *Gillisia* genus (1.4%, representing 7.7% of the phylum), in which members have been detected in cold saline (Dorador et al., 2009; Maida et al., 2014) and athalassohaline (Montoya et al., 2013) environments. The pangenome of the *Gillisia* genus reveals the presence of genes involved in the adaptation to cold and high salinity (Maida et al., 2014). The *Geothermobacter* (2.3%) and *Desulfovermiculus* (2.2%) were the dominant genera of the Desulfobacterota, representing, respectively, 19.1 and 18.1% of the phylum. The *Geothermobacter* genus, iron-reducing bacterium (Gomez-Saez et al., 2017), and the *Desulfovermiculus*, sulfate-reducing bacterium (Nigro et al., 2020) are usually found in hypersaline environments where they play a key role in iron and sulfur cycling. The archaeal phylum Halobacteriota was dominated by *Halapricum* (6.1%), *Halorubrum* (3.6%), and *Natronomonas* (2.2%) genera (representing, respectively, 12, 7, and 4.3% of the phylum), isolated in hypersaline ecosystems where they often represent the dominant archaeal taxa (Tu et al., 2022). *Halorubrum* and *Natronomonas* are known as aerobes (Mora-Ruiz et al., 2018), while *Halapricum* is a sulfate-reducing archaeon (Sorokin et al., 2022). Beside these genera from the dominant phyla, it is worth noting the presence of *Halanaerobium* (8.3%, representing 83.4% of the *Halanaerobiaeota*) and *Truepera* (8.5%, representing 99.8% of the *Deinococcota*), chemoorganotrophic strictly anaerobic and aerobic bacteria, respectively (Oren, 2015; Albuquerque et al., 2018), which are detected in hypersaline ecosystems (Boidi et al., 2022; Solchaga et al., 2022). Therefore, such observation indicated that the microbial community inhabiting the Salineta wetland is characteristic of extreme saline environments.

The comparison of microbial communities from the different landscapes revealed that layer (depth) was the main factor explaining the microbial distribution (PERMANOVA: *R*^2^ = 0.2, *p* < 0.001). The layers were identified according to soil colors, which rely on chemical and physical characteristics (Kalev and Toor, 2018), particularly the ORP gradient from low (ORP, −155 ± 193 mV) at the surface to higher redox (ORP, 88 ± 118 mV) in the deeper layer. Such microbial distribution according to depth was in accordance with previous reports, showing a relationship between microbial community structure and the variation of physical–chemical parameters with depth in microbial mats (Fourçans et al., 2004) and marine sediments (Böer et al., 2009). Furthermore, the microbial biomarkers revealed by LEfSe were affiliated with five bacterial OTUs (belonging to the Anaerolineaceae family and the *Salinarimonas*, *Rhodopirellula*, *Desulfonatronobacter*, and *Aliifodinibius* genera) and two archaeal OTUs (*Halorubellus* genus and *H. formicicum*) for the upper layer. Consistent with low ORP observed at the upper layer L1, these OTUs are affiliated with taxa detected in hypersaline ecosystems exhibiting strictly (Anaerolineaceae, *Desulfonatronobacter*, *Aliifodinibius*, *H. formicicum*) or facultative (*Salinarimonas*, *Rhodopirellula*) anaerobic metabolisms (Cai et al., 2011; Cui et al., 2012; d’Avó et al., 2013; Sorokin et al., 2015, 2017; Sugihara et al., 2016). They have also been shown to possess adaptation capacities to sudden environmental changes (Wecker et al., 2009; Cui et al., 2012) that might explain their adaptation to the extreme and fluctuating environmental conditions prevailing at Salineta wetland, especially at the surface soil layer. Interestingly, members of the Anaerolineaceae have been found associated with methanogens (Liang et al., 2015), further supporting that the surface L1 layer was dominated by anaerobic metabolisms.

For the deeper layers, the biomarkers revealed by LEfSe analysis correspond to OTUs affiliated with halophilic bacteria found in hypersaline ecosystems. They include *Coxiella*, *Anaerophaga*, and Balneolaceae for layer L2, exhibiting anaerobic metabolism (Song et al., 2021; Wu et al., 2022). This finding is in agreement with the reversed redox gradient observed in the Salineta wetland. The dominance of *Coxiella*, a genus of endosymbiont bacteria pathogenic for humans and animals (Lory, 2014), is surprising, although the presence of *Coxiella* has been linked to cyanobacterial and algal blooms (Li et al., 2011) in agreement with their lifestyle. Furthermore, members of the *Coxiella* genus have been detected in seawater associated with urban wastewater inputs (Fonti et al., 2021). The farming activities around the Salineta wetland and the presence of migratory birds represent potential sources of *Coxiella* since it has been detected in the feces of livestock (Mcquiston and Childs, 2002) and wild birds (Ebani and Mancianti, 2022).

Linear discriminant analysis effect size also identified *Haloplanus* and *Halonotius* as microbial biomarkers for the halophytes vegetated soil (station S3). *Haloplanus* and *Halonotius* are extremely halophilic archaea, members of which have been isolated from various hypersaline environments (Elevi Bardavid et al., 2007; Burns et al., 2010; Cui et al., 2010). It is likely that the OTUs identified as biomarkers for station S3’s own metabolism well adapted to the environmental fluctuation, including variations in salinity, temperature, and UV radiation (Youssef et al., 2012). Finally, LEfSe revealed different biomarkers according to seasons, reflecting probably the variations of not only salinity according to wet and dry periods but also temperature and sun irradiance. The biomarkers taxa have been detected in hypersaline ecosystems: *Candidatus*, *Halobonum*, and *Halanaerobium*, archaeon and sulfidogenic bacterium, respectively (Abdeljabbar et al., 2013; Ugalde et al., 2013) for winter (wet period), and *Desulfurivibrio*, sulfidogenic bacterium (Sorokin et al., 2008) for summer (dry period). The dominance of sulfidogenic bacteria in both seasons is in agreement with the fact that the microbial sulfur cycle is among the most active in alkaline hypersaline lakes (Sorokin et al., 2011). The fact that different taxa ensure similar functions according to seasons suggests functional redundancy.

The PICRUSt2 functional capabilities of microbial communities inferred from 16S rRNA gene meta-barcoding data revealed similar functional patterns, irrespective of the layer, the landscape type, or the season. Consistently, the functional distribution within the microbial community assessed by PCA was explained by the presence of different KOs for each functional group. Such observations suggest functional redundancy; however, further analyses are requested to understand the adaptation strategies for maintaining biogeochemical cycle functioning. Future research will benefit from functional untargeted metagenomic analyses to understand microbial functions in the Salineta hypersaline wetland. Functional redundancy was observed in many ecosystems (Staley et al., 2016; Louca et al., 2018), allowing the microbial community to cope with fluctuating environmental conditions (Stauffert et al., 2014; Terrisse et al., 2017).

Sulfur cycle functions were positively correlated with nitrogen cycle functions (denitrification, DNRA, and COMAMMOX) but not significantly correlated with nitrogen fixation and nitrification. Nitrogen and sulfur cycles are important for ecosystem functioning, and they are coupled in aquatic sediment (Zhu et al., 2018). Our results suggest that the sulfur cycle affects the nitrogen metabolism, some reactions involved in nitrogen species transformation, but not the overall nitrogen cycle functioning. The functions involved in the sulfur cycle were also positively correlated with the functions of the carbon cycle involved in carbon fixation and methane metabolism. Noteworthy, methane metabolism functions (methanogenesis and methanotrophy) were abundant on the surface layer exhibiting the lowest ORP, particularly in the usually submerged soil (station S1). In general, the microorganisms involved in methane metabolisms are described in depth layers with negative redox (Jørgensen, 1982; Kristensen, 2000), competing for carbon sources or establishing syntrophic interactions (Schink and Stams, 2006). Our results suggest that in the reverse redox gradient, methanogens and sulfate reducers are present at the upper layer of the soil, being more abundant during the wet season (winter) showing the effect of flooding on microbial functions. The coexistence of methanogens and sulfate reducers has been described in various ecosystems, such as saline coastal areas (Egger et al., 2016), salt marshes (Parkes et al., 2012), and athalassohaline lakes (Montoya et al., 2011). It has been shown that methanogenesis and sulfate reduction can be coupled even at elevated sulfate concentrations, the competition for a common substrate controlling the activities (Sela-Adler et al., 2017). However, factors favoring sulfate reduction, such as increasing salinity (Huang et al., 2020), resulting in decreasing methane fluxes (McGenity, 2010; Marton et al., 2012). In contrast, the input of freshwater from flooding events reducing salinity favors methane fluxes (McGenity, 2010) and greenhouse gas emissions (Helton et al., 2018). It is likely that climate change with more frequent rainfalls accompanied by heavy flooding events as well as human activities such as intensive agriculture involving important irrigation threatens the equilibrium of ephemeral saline lakes by modifying particularly redox gradient, which, in turn, affects greenhouse gas emissions. Thus, the Salineta wetland represents an adequate model to study the effects of climate change on saline wetlands that would provide information on microbial community modifications and their ecosystem services.

## Conclusion

The three sampling stations in the Salineta wetland, within a gradient of water presence (S1 soil usually submerged, S2 soil intermittently flooded, and S3 covered by halophytes), were all characterized by reversed redox gradient in the soil profile (increasing from surface to subsurface soil layer). Thus, the soil layers were the main drivers explaining microbial diversity distribution. Although they exhibited different taxonomic microbial communities, the predictive functional analysis revealed similar functions involved in the main biogeochemical cycles (nitrogen, sulfur, and carbon) in all the soil layers, suggesting functional redundancy. It is likely that the microbial communities inhabiting the Salineta wetland are well adapted to the extreme environmental conditions, characterized by high salinity, high radiance, and seasonal drying. The functioning in this highly dynamic ecosystem is ensured by the redundancy of the biogeochemical cycle functions. Our study provides the first microbial inventory of the Salineta wetland, which is threatened by the direct input of agrochemicals and freshwater from the surrounding irrigated areas. As a consequence, the water change to less saline and polluted may modify the environmental parameters including the redox gradient, which, in turn, affect microbial communities dramatically disturbing the ecosystem. The information from our results is then useful to monitor and manage such an extreme wetland exposed to human activities.

## Data availability statement

The datasets presented in this study can be found in online repositories. The names of the repository/repositories and accession number PRJNA763109 can be found in the article/Supplementary material.

## Author contributions

ZB conducted experimental and performed the bioinformatics and biostatistics analyses. RR-O and JÁ-F conducted the physical–chemical analyses. ZB, CaC, ChC, CC-L, and RD participated in the conceptualization, validation, and writing. All authors contributed to the article and approved the submitted version.

## Footnotes

1
https://www.arb-silva.de/search/testprime

2
https://github.com/lanzen/CREST

3
http://www.rproject.org

## References

[B1] Abdeljabbar H. Cayol J. L. Ben Hania W. Boudabous A. Sadfi N. Fardeau M. L. (2013). *Halanaerobium sehlinense* sp. nov., an extremely halophilic, fermentative, strictly anaerobic bacterium from sediments of the hypersaline lake Sehline Sebkha. *Int. J. Syst. Evol. Microbiol.* 63 2069–2074. 10.1099/ijs.0.040139-0 23064350

[B2] Albuquerque L. Rainey F. A. da Costa M. S. (2018). “Truepera,” in *Bergey’s manual of systematics of archaea and bacteria*, eds Trujillo M. E. Dedysh S. DeVos P. Hedlund B. Kämpfer P. Rainey F. A. (Hoboken, NJ: John Wiley & Sons). 10.1002/9781118960608.gbm01328

[B3] Andrei A. Ş Banciu H. L. Oren A. (2012). Living with salt: Metabolic and phylogenetic diversity of archaea inhabiting saline ecosystems. *FEMS Microbiol. Lett.* 330 1–9. 10.1111/j.1574-6968.2012.02526.x 22339687

[B4] Arce-Rodríguez A. Puente-Sánchez F. Avendaño R. Libby E. Mora-Amador R. Rojas-Jimenez K. (2020). Microbial community structure along a horizontal oxygen gradient in a Costarican volcanic influenced acid rock drainage system. *Microb. Ecol.* 80 793–808. 10.1007/s00248-020-01530-9 32572534

[B5] Ben Salem F. Ben Said O. Aissa P. Mahmoudi E. Monperrus M. Grunberger O. (2016). Pesticides in Ichkeul Lake–Bizerta Lagoon watershed in Tunisia: Use, occurrence, and effects on bacteria and free-living marine nematodes. *Environ. Sci. Pollut. R.* 23 36–48. 10.1007/S11356-015-4991-8 26165992

[B6] Böer S. I. Hedtkamp S. I. C. van Beusekom J. E. E. Fuhrman J. A. Boetius A. Ramette A. (2009). Time–and sediment depth-related variations in bacterial diversity and community structure in subtidal sands. *ISME J.* 3 780–791. 10.1038/ismej.2009.29 19340087

[B7] Boidi F. J. Mlewski E. C. Fernández G. C. Flores M. R. Gérard E. Farías M. E. (2022). Community vertical composition of the laguna Negra hypersaline microbial mat, Puna region (Argentinean Andes). *Biology (Basel)* 11:831. 10.3390/biology11060831 35741352 PMC9220024

[B8] Bolhuis H. Cretoiu M. S. Stal L. J. (2014). Molecular ecology of microbial mats. *FEMS Microbiol. Ecol.* 90 335–350. 10.1111/1574-6941.12408 25109247

[B9] Bordenave S. Fourçans A. Blanchard S. Goñi M. S. Duran R. (2004). Structure and functional analyses of bacterial communities changes in microbial mats following petroleum exposure. *Ophelia* 58 195–203. 10.1080/00785236.2004.10410227

[B10] Borsodi A. K. Mucsi M. Krett G. Szabó A. Felföldi T. Szili-Kovács T. (2021). Variation in sodic soil bacterial communities associated with different alkali vegetation types. *Microorganisms* 9:1673. 10.3390/microorganisms9081673 34442752 PMC8402138

[B11] Bourhane Z. Lanzén A. Cagnon C. Ben Said O. Mahmoudi E. Coulon F. (2022). Microbial diversity alteration reveals biomarkers of contamination in soil-river-lake continuum. *J. Hazard. Mater.* 421:126789. 10.1016/j.jhazmat.2021.126789 34365235

[B12] Bruneel O. Pascault N. Egal M. Bancon-Montigny C. Goñi-Urriza M. S. Elbaz-Poulichet F. (2008). Archaeal diversity in a Fe-As rich acid mine drainage at Carnoulès (France). *Extremophiles* 12 563–571. 10.1007/s00792-008-0160-z 18418543

[B13] Burns D. G. Janssen P. H. Itoh T. Kamekura M. Echigo A. Dyall-Smith M. L. (2010). *Halonotius pteroides* gen. nov., sp. nov., an extremely halophilic archaeon recovered from a saltern crystallizer in southern Australia. *Int. J. Syst. Evol. Microbiol.* 60 1196–1199. 10.1099/ijs.0.010017-0 19667389

[B14] Cai M. Wang L. Cai H. Li Y. Wang Y. N. Tang Y. Q. (2011). *Salinarimonas ramus* sp. nov. and *Tessaracoccus oleiagri* sp. nov., isolated from a crude oil-contaminated saline soil. *Int. J. Syst. Evol. Microbiol.* 61 1767–1775. 10.1099/ijs.0.025932-0 20802058

[B15] Casamayor E. O. Triadó-Margarit X. Castañeda C. (2013). Microbial biodiversity in saline shallow lakes of the Monegros Desert, Spain. *FEMS Microbiol. Ecol.* 85 503–518. 10.1111/1574-6941.12139 23621854

[B16] Castañeda C. García-Vera M. Á (2008). Water balance in the playa-lakes of an arid environment, Monegros, NE Spain. *Hydrogeol. J.* 16 87–102. 10.1007/s10040-007-0230-9

[B17] Castañeda C. Herrero J. (2005). The water regime of the Monegros playa-lakes as established from ground and satellite data. *J. Hydrol. (Amst)* 310 95–110. 10.1016/J.JHYDROL.2004.12.007

[B18] Castañeda C. Javier Gracia F. Luna E. Rodríguez-Ochoa R. (2015). Edaphic and geomorphic evidences of water level fluctuations in Gallocanta Lake, NE Spain. *Geoderma* 23 265–279. 10.1016/j.geoderma.2014.11.005

[B19] Castañeda C. Luna E. Rabenhorst M. (2017). Reducing conditions in soil of Gallocanta Lake, northeast Spain. *Eur. J. Soil Sci.* 68 249–258. 10.1111/ejss.12407

[B20] Caton I. R. Schneegurt M. A. (2012). Culture-independent analysis of the soil bacterial assemblage at the Great Salt Plains of Oklahoma. *J. Basic Microbiol.* 52 16–26. 10.1002/jobm.201100175 21953014 PMC3597346

[B21] Conesa J. A. Castañeda C. Pedrol J. (2011). *Las saladas de Monegros y su entorno: Habitats y paisaje vegetal.* Zaragoza: Consejo de Protección de la Naturaleza de Aragón.

[B22] Cui H. L. Mou Y. Z. Yang X. Zhou Y. G. Liu H. C. Zhou P. J. (2012). *Halorubellus salinus* gen. nov., sp. nov. and *Halorubellus litoreus* sp. nov., novel halophilic archaea isolated from a marine solar saltern. *Syst. Appl. Microbiol.* 35 30–34. 10.1016/j.syapm.2011.08.001 21889861

[B23] Cui H.-L. Gao X. Li X.-Y. Xu X.-W. Zhou Y.-G. Liu H.-C. (2010). *Haloplanus vescus* sp. nov., an extremely halophilic archaeon from a marine solar saltern, and emended description of the genus *Haloplanus*. *Int. J. Syst. Evol. Microbiol.* 60 1824–1827. 10.1099/ijs.0.018564-0 19767368

[B24] Cycil L. M. DasSarma S. Pecher W. McDonald R. AbdulSalam M. Hasan F. (2020). Metagenomic insights into the diversity of halophilic microorganisms indigenous to the Karak salt mine, Pakistan. *Front. Microbiol.* 11:1567. 10.3389/fmicb.2020.01567 32793134 PMC7386132

[B25] d’Avó A. F. Cunha S. Mingote A. Lamosa P. da Costa M. S. Costa J. (2013). A unique pool of compatible solutes on *Rhodopirellula baltica*, member of the deep-branching phylum Planctomycetes. *PLoS One* 8:e68289. 10.1371/journal.pone.0068289 23826385 PMC3694870

[B26] Demergasso C. Escudero L. Casamayor E. O. Chong G. Balagué V. Pedrós-Alió C. (2008). Novelty and spatio-temporal heterogeneity in the bacterial diversity of hypersaline Lake Tebenquiche (Salar de Atacama). *Extremophiles* 12 491–504. 10.1007/s00792-008-0153-y 18347752

[B27] Dominguez-Beisiegel M. Herrero J. Castañeda C. (2011). Saline wetlands’ fate in inland deserts: An example of 80 years’ decline from Monegros, Spain. *Land Degrad. Dev.* 24 250–265. 10.1002/ldr.1122

[B28] Dong H. Zhang G. Jiang H. Yu B. Chapman L. R. Lucas C. R. (2006). Microbial diversity in sediments of saline Qinghai Lake, China: Linking geochemical controls to microbial ecology. *Microb. Ecol.* 51 65–82. 10.1007/s00248-005-0228-6 16400537

[B29] Dorador C. Meneses D. Urtuvia V. Demergasso C. Vila I. Witzel K. P. (2009). Diversity of bacteroidetes in high-altitude saline evaporitic basins in northern Chile. *J. Geophys. Res. Biogeosci.* 114:11. 10.1029/2008JG000837

[B30] Douglas G. M. Maffei V. J. Zaneveld J. Yurgel S. N. Brown J. R. Taylor C. M. (2020). PICRUSt2: An improved and customizable approach for metagenome inference. *bioRxiv* [Preprint]. 10.1101/672295

[B31] Dufresne Y. Lejzerowicz F. Perret-Gentil L. A. Pawlowski J. Cordier T. (2019). SLIM: A flexible web application for the reproducible processing of environmental DNA metabarcoding data. *BMC Bioinform.* 20:88. 10.1186/s12859-019-2663-2 30782112 PMC6381720

[B32] Duran R. Cravo-Laureau C. (2016). Role of environmental factors and microorganisms in determining the fate of polycyclic aromatic hydrocarbons in the marine environment. *FEMS Microbiol. Rev.* 40 814–830. 10.1093/femsre/fuw031 28201512 PMC5091036

[B33] Ebani V. V. Mancianti F. (2022). Potential role of birds in the epidemiology of *Coxiella burnetii*, *Coxiella*-like agents and *Hepatozoon* spp. *Pathogens* 11:298. 10.3390/pathogens11030298 35335622 PMC8954922

[B34] Edgar R. C. (2016). UNCROSS: Filtering of high-frequency cross-talk in 16S amplicon reads. *bioRxiv* [Preprint]. 10.1101/088666

[B35] Egger M. Lenstra W. Jong D. Meysman F. J. R. Sapart C. J. van der Veen C. (2016). Rapid sediment accumulation results in high methane effluxes from coastal sediments. *PLoS One* 11:e0161609. 10.1371/journal.pone.0161609 27560511 PMC4999275

[B36] Elbrecht V. Vamos E. E. Steinke D. Leese F. (2018). Estimating intraspecific genetic diversity from community DNA metabarcoding data. *PeerJ* 2018 1–13. 10.7717/peerj.4644 29666773 PMC5896493

[B37] Elevi Bardavid R. Mana L. Oren A. (2007). *Haloplanus natans* gen. nov., sp. nov., an extremely halophilic, gas-vacuolate archaeon isolated from dead sea-red sea water mixtures in experimental outdoor ponds. *Int. J. Syst. Evol. Microbiol.* 57 780–783. 10.1099/ijs.0.64648-0 17392206

[B38] Fonti V. di Cesare A. Šangulin J. del Negro P. Celussi M. (2021). Antibiotic resistance genes and potentially pathogenic bacteria in the central Adriatic Sea: Are they connected to urban wastewater inputs? *Water (Switzerland)* 13:3335. 10.3390/w13233335

[B39] Fourçans A. de Oteyza T. G. Wieland A. Solé A. Diestra E. van Bleijswijk J. (2004). Characterization of functional bacterial groups in a hypersaline microbial mat community (Salins-de-Giraud, Camargue, France). *FEMS Microbiol. Ecol.* 51 55–70. 10.1016/j.femsec.2004.07.012 16329855

[B40] Franco-Luesma S. Cavero J. Plaza-Bonilla D. Cantero-Martínez C. Arrúe J. L. Álvaro-Fuentes J. (2020). Tillage and irrigation system effects on soil carbon dioxide (CO2) and methane (CH4) emissions in a maize monoculture under Mediterranean conditions. *Soil Tillage Res.* 196:104488. 10.1016/j.still.2019.104488

[B41] Frøslev T. G. Kjøller R. Bruun H. H. Ejrnæs R. Brunbjerg A. K. Pietroni C. (2017). Algorithm for post-clustering curation of DNA amplicon data yields reliable biodiversity estimates. *Nat. Commun.* 8:1188. 10.1038/s41467-017-01312-x 29084957 PMC5662604

[B42] Gasol J. M. Casamayor E. O. Joint I. Garde K. Gustavson K. Benlloch S. (2004). Control of heterotrophic prokaryotic abundance and growth rate in hypersaline planktonic environments. *Aqua. Microb. Ecol.* 34 193–206. 10.3354/ame034193

[B43] Giloteaux L. Duran R. Casiot C. Bruneel O. Elbaz-Poulichet F. Goñi-Urriza M. (2013). Three-year survey of sulfate-reducing bacteria community structure in Carnoulès acid mine drainage (France), highly contaminated by arsenic. *FEMS Microbiol. Ecol.* 83 724–737. 10.1111/1574-6941.12028 23057444

[B44] Gomez-Saez G. V. Ristova P. P. Sievert S. M. Elvert M. Hinrichs K. U. Bühring S. I. (2017). Relative importance of chemoautotrophy for primary production in a light exposed marine shallow hydrothermal system. *Front. Microbiol.* 8:702. 10.3389/fmicb.2017.00702 28484442 PMC5399606

[B45] Helton A. M. Ardón M. Bernhardt E. S. (2018). Hydrologic context alters greenhouse gas feedbacks of coastal wetland salinization. *Ecosystems* 22 1108–1125. 10.1007/s10021-018-0325-2

[B46] Huang J. Yang J. Jiang H. Wu G. Liu W. Wang B. (2020). Microbial responses to simulated salinization and desalinization in the sediments of the Qinghai–Tibetan lakes. *Front. Microbiol.* 11:1772. 10.3389/fmicb.2020.01772 32849396 PMC7426462

[B47] Jiang H. Dong H. Zhang G. Yu B. Chapman L. R. Fields M. W. (2006). Microbial diversity in water and sediment of Lake Chaka, an athalassohaline lake in northwestern China. *Appl. Environ. Microbiol.* 72 3832–3845. 10.1128/AEM.02869-05 16751487 PMC1489620

[B48] Jørgensen B. B. (1982). Mineralization of organic matter in the sea bed–The role of sulphate reduction. *Nature* 296 643–645. 10.1038/296643a0

[B49] Kalev S. D. Toor G. S. (2018). “The composition of soils and sediments,” in *Green chemistry: An inclusive approach*, eds Török B. Dransfield T. (Amsterdam: Elsevier), 339–357. 10.1016/B978-0-12-809270-5.00014-5

[B50] Kan J. Wang K. Chen F. (2006). Temporal variation and detection limit of an estuarine bacterioplankton community analyzed by denaturing gradient gel electrophoresis (DGGE). *Aquat. Microb. Ecol.* 42 7–18. 10.3354/ame042007

[B51] Karner M. B. Delong E. F. Karl D. M. (2001). Archaeal dominance in the mesopelagic zone of the Pacific Ocean. *Nature* 409 507–510. 11206545 10.1038/35054051

[B52] Kirk Harris J. Gregory Caporaso J. Walker J. J. Spear J. R. Gold N. J. Robertson C. E. (2013). Phylogenetic stratigraphy in the Guerrero Negro hypersaline microbial mat. *ISME J.* 7 50–60. 10.1038/ismej.2012.79 22832344 PMC3526174

[B53] Kristensen E. (2000). Organic matter diagenesis at the oxic/anoxic interface in coastal marine sediments, with emphasis on the role of burrowing animals. *Hydrobiologia* 426 1–24. 10.1023/A:1003980226194

[B54] Kroeger K. D. Crooks S. Moseman-Valtierra S. Tang J. (2017). Restoring tides to reduce methane emissions in impounded wetlands: A new and potent blue carbon climate change intervention. *Sci. Rep.* 7 1–12. 10.1038/s41598-017-12138-4 28931842 PMC5607314

[B55] Langille M. G. I. Zaneveld J. Caporaso J. G. McDonald D. Knights D. Reyes J. A. (2013). Predictive functional profiling of microbial communities using 16S rRNA marker gene sequences. *Nat. Biotechnol.* 31 814–821. 10.1038/nbt.2676 23975157 PMC3819121

[B56] Lanzén A. Jørgensen S. L. Huson D. H. Gorfer M. Grindhaug S. H. Jonassen I. (2012). CREST - classification resources for environmental sequence tags. *PLoS One* 7:e49334. 10.1371/journal.pone.0049334 23145153 PMC3493522

[B57] Lanzén A. Mendibil I. Borja Á Alonso-Sáez L. (2021). A microbial mandala for environmental monitoring: Predicting multiple impacts on estuarine prokaryote communities of the Bay of Biscay. *Mol. Ecol.* 30 2969–2987. 10.1111/mec.15489 32479653

[B58] Lanzén A. Simachew A. Gessesse A. Chmolowska D. Jonassen I. Øvreås L. (2013). Surprising prokaryotic and eukaryotic diversity, community structure and biogeography of Ethiopian Soda Lakes. *PLoS One* 8:e72577. 10.1371/journal.pone.0072577 24023625 PMC3758324

[B59] Li H. Xing P. Chen M. Bian Y. Wu Q. L. (2011). Short-term bacterial community composition dynamics in response to accumulation and breakdown of microcystis blooms. *Water Res.* 45 1702–1710. 10.1016/j.watres.2010.11.011 21185055

[B60] Liang B. Wang L. Y. Mbadinga S. M. Liu J. F. Yang S. Z. Gu J. D. (2015). *Anaerolineaceae* and *Methanosaeta* turned to be the dominant microorganisms in alkanes-dependent methanogenic culture after long-term of incubation. *AMB Express* 5:37. 10.1186/s13568-015-0117-4 26080793 PMC4469597

[B61] Lory S. (2014). “The family Coxiellaceae,” in *The prokaryotes*, eds Rosenberg E. DeLong E. F. Lory S. Stackebrandt E. Thompson F. (Berlin: Springer). 10.1007/978-3-642-38922-1_371

[B62] Louca S. Polz M. F. Mazel F. Albright M. B. N. Huber J. A. O’Connor M. I. (2018). Function and functional redundancy in microbial systems. *Nat. Ecol. Evol.* 2 936–943. 10.1038/s41559-018-0519-1 29662222

[B63] Mahé F. Rognes T. Quince C. de Vargas C. Dunthorn M. (2015). Swarmv2: Highly-scalable and high-resolution amplicon clustering. *PeerJ* 3:e1420. 10.7717/peerj.1420 26713226 PMC4690345

[B64] Maida I. Fondi M. Papaleo M. C. Perrin E. Orlandini V. Emiliani G. (2014). Phenotypic and genomic characterization of the Antarctic bacterium *Gillisia* sp. CAL575, a producer of antimicrobial compounds. *Extremophiles* 18 35–49. 10.1007/s00792-013-0590-0 24150693

[B65] Martin M. (2011). Cutadapt removes adapter sequences from high-throughput sequencing reads. *EMBnet J.* 17 10–12. 10.14806/ej.17.1.200

[B66] Marton J. M. Herbert E. R. Craft C. B. (2012). Effects of salinity on denitrification and greenhouse gas production from laboratory-incubated tidal forest soils. *Wetlands* 32 347–357. 10.1007/s13157-012-0270-3

[B67] Mau R. L. Liu C. M. Aziz M. Schwartz E. Dijkstra P. Marks J. C. (2015). Linking soil bacterial biodiversity and soil carbon stability. *ISME J.* 9 1477–1480. 10.1038/ismej.2014.205 25350158 PMC4438316

[B68] Mazière C. Agogué H. Cravo-Laureau C. Cagnon C. Lanneluc I. Sablé S. (2021). New insights in bacterial and eukaryotic diversity of microbial mats inhabiting exploited and abandoned salterns at the Ré Island (France). *Microbiol Res.* 252:126854. 10.1016/j.micres.2021.126854 34454310

[B69] McGenity T. (2010). “Methanogens and methanogenesis in hypersaline environments,” in *Handbook of hydrocarbon and lipid microbiology*, ed. Timmis K. N. (Berlin: Springer). 10.1007/978-3-540-77587-4_53

[B70] McGenity T. Oren A. (2012). “Hypersaline environments,” in *Life at extremes: Environments, organisms and strategies for survival*, ed. Bell E. (Cambridge, MA: Cabi), 402–437.

[B71] Mcquiston J. H. Childs J. E. (2002). Review Q fever in humans and animals in the United States. *Vector Borne Zoonotic Dis.* 2 179–191. 12737547 10.1089/15303660260613747

[B72] Mehrshad M. Amoozegar M. A. Didari M. Bagheri M. Shahzadeh Fazeli S. A. Schumann P. (2013). *Bacillus halosaccharovorans* sp. nov., a moderately halophilic bacterium from a hypersaline lake. *Int. J. Syst. Evol. Microbiol.* 63 2776–2781. 10.1099/ijs.0.046961-0 23291894

[B73] Menéndez-Serra M. Triadó-Margarit X. Casamayor E. O. (2021). Ecological and metabolic thresholds in the bacterial, protist, and fungal microbiome of ephemeral saline lakes (Monegros Desert, Spain). *Microb. Ecol.* 82 885–896. 10.1007/s00248-021-01732-9 33725151

[B74] Menéndez-Serra M. Triadó-Margarit X. Castañeda C. Herrero J. Casamayor E. O. (2019). Microbial composition, potential functional roles and genetic novelty in gypsum-rich and hypersaline soils of Monegros and Gallocanta (Spain). *Sci. Total Environ.* 650 343–353. 10.1016/j.scitotenv.2018.09.050 30199680

[B75] Montoya L. Lozada-Chávez I. Amils R. Rodriguez N. Marín I. (2011). The sulfate-rich and extreme saline sediment of the ephemeral Tirez Lagoon: A biotope for acetoclastic sulfate-reducing bacteria and hydrogenotrophic methanogenic archaea. *Int. J. Microbiol.* 2011:753758. 10.1155/2011/753758 21915180 PMC3170894

[B76] Montoya L. Vizioli C. Rodríguez N. Rastoll M. J. Amils R. Marin I. (2013). Microbial community composition of Tirez Lagoon (Spain), a highly sulfated athalassohaline environment. *Aquat. Biosyst.* 9:19. 10.1186/2046-9063-9-19 24083554 PMC3852488

[B77] Mora-Ruiz M. del R. Cifuentes A. Font-Verdera F. Pérez-Fernández C. Farias M. E. (2018). Biogeographical patterns of bacterial and archaeal communities from distant hypersaline environments. *Syst. Appl. Microbiol.* 41 139–150. 10.1016/j.syapm.2017.10.006 29352612

[B78] Nigro L. M. Elling F. J. Hinrichs K. U. Joye S. B. Teske A. (2020). Microbial ecology and biogeochemistry of hypersaline sediments in Orca Basin. *PLoS One* 15:e0231676. 10.1371/journal.pone.0231676 32315331 PMC7173876

[B79] Offre P. Spang A. Schleper C. (2013). Archaea in biogeochemical cycles. *Annu. Rev. Microbiol.* 67 437–457. 10.1146/annurev-micro-092412-155614 23808334

[B80] Oksanen A. J. Blanchet F. G. Friendly M. Kindt R. Legendre P. McGlinn D. (2019). *Vegan: Community ecology package. R package version 2.2-0.*

[B81] Oren A. (2013). “Life in magnesium–and calcium-rich hypersaline environments: Salt stress by chaotropic ions,” in *Polyextremophiles. Cellular origin, life in extreme habitats and astrobiology*, Vol. 27 eds Seckbach J. Oren A. Stan-Lotter H. (Dordrecht: Springer). 10.1007/978-94-007-6488-0_8

[B82] Oren A. (2015). “Life in high-salinity environments,” in *Manual of environmental microbiology*, eds Yates M. Nakatsu C. Miller R. Pillai S. (Washington, DC: ASM Press). 10.1128/9781555818821.ch4.3.2

[B83] Pagaling E. Wang H. Venables M. Wallace A. Grant W. D. Cowan D. A. (2009). Microbial biogeography of six salt lakes in Inner Mongolia, China, and a salt lake in Argentina. *Appl. Environ. Microbiol.* 75 5750–5760. 10.1128/AEM.00040-09 19648369 PMC2747855

[B84] Parkes R. J. Brock F. Banning N. Hornibrook E. R. Roussel E. G. Weightman A. J. (2012). Changes in methanogenic substrate utilization and communities with depth in a salt-marsh, creek sediment in southern England. *Estuar Coast Shelf Sci.* 96 170–178. 10.1016/j.ecss.2011.10.025

[B85] Ramsar Convention Secretariat (2010). *Designating Ramsar sites: Strategic framework and guidelines for the future development of the list of wetlands of international importance. Ramsar handbooks for the wise use of wetlands.* Gland: Ramsar Convention Secretariat.

[B86] Reddy K. R. DeLaune R. D. (2008). Biogeochemistry of wetlands: Science and applications. *Biogeochem. Wetlands Sci. Appl.* 73:692. 10.2136/sssaj2008.0013br

[B87] Ringeval B. de Noblet-Ducoudré N. Ciais P. Bousquet P. Prigent C. Papa F. (2010). An attempt to quantify the impact of changes in wetland extent on methane emissions on the seasonal and interannual time scales. *Glob. Biogeochem. Cycles* 24 1–12. 10.1029/2008GB003354

[B88] Rognes T. Flouri T. Nichols B. Quince C. Mahé F. (2016). VSEARCH: A versatile open source tool for metagenomics. *PeerJ* 4:e2548. 10.7717/peerj.2584 27781170 PMC5075697

[B89] Schink B. Stams A. J. M. (2006). “Syntrophism among prokaryotes,” in *The prokaryotes*, eds Dworkin M. Falkow S. Rosenberg E. Schleifer K. Stackebrandt E. (New York, NY: Springer), 309–335. 10.1007/0-387-30742-7_11

[B90] Schultz P. W. (2000). Empathizing with nature: The effects of perspective taking on concern for environmental issues. *J. Soc. Issues* 56 391–406. 10.1111/0022-4537.00174

[B91] Segata N. Izard J. Waldron L. Gevers D. Miropolsky L. Garrett W. S. (2011). Metagenomic biomarker discovery and explanation. *Genome Biol.* 12:R60. 10.1186/gb-2011-12-6-r60 21702898 PMC3218848

[B92] Sela-Adler M. Ronen Z. Herut B. Antler G. Vigderovich H. Eckert W. (2017). Co-existence of methanogenesis and sulfate reduction with common substrates in sulfate-rich estuarine sediments. *Front. Microbiol.* 8:766. 10.3389/fmicb.2017.00766 28529500 PMC5418336

[B93] Solchaga J. I. Busalmen J. P. Nercessian D. (2022). Unraveling anaerobic metabolisms in a hypersaline sediment. *Front. Microbiol.* 13:811432. 10.3389/fmicb.2022.811432 35369499 PMC8966722

[B94] Song Q. Chen X. Zhou W. Xie X. (2021). Application of a spiral symmetric stream anaerobic bioreactor for treating saline heparin sodium pharmaceutical wastewater: Reactor operating characteristics, organics degradation pathway and salt tolerance mechanism. *Water Res.* 205:117671. 10.1016/j.watres.2021.117671 34555740

[B95] Sorokin D. Y. Chernyh N. A. Poroshina M. N. (2015). *Desulfonatronobacter acetoxydans* sp. nov.,: A first acetate-oxidizing, extremely salt-tolerant alkaliphilic SRB from a hypersaline soda lake. *Extremophiles* 19 899–907. 26085472 10.1007/s00792-015-0765-y PMC4546703

[B96] Sorokin D. Y. Kuenen J. G. Muyzer G. (2011). The microbial sulfur cycle at extremely haloalkaline conditions of soda lakes. *Front. Microbiol.* 2:44. 10.3389/fmicb.2011.00044 21747784 PMC3128939

[B97] Sorokin D. Y. Merkel A. Y. Messina E. Tugui C. Pabst M. Golyshin P. N. (2022). Anaerobic carboxydotrophy in sulfur-respiring haloarchaea from hypersaline lakes. *ISME J.* 16 1534–1546. 10.1038/s41396-022-01206-x 35132120 PMC9123189

[B98] Sorokin D. Y. Messina E. Smedile F. Roman P. Damste J. S. Ciordia S. (2017). Discovery of anaerobic lithoheterotrophic haloarchaea, ubiquitous in hypersaline habitats. *ISME J.* 11 1245–1260. 10.1038/ismej.2016.203 28106880 PMC5437934

[B99] Sorokin D. Y. Mosier D. Zorz J. K. Dong X. Strous M. (2020). Wenzhouxiangella strain AB-CW3, a proteolytic bacterium from hypersaline soda lakes that preys on cells of gram-positive bacteria. *Front. Microbiol.* 11:597686. 10.3389/fmicb.2020.597686 33281797 PMC7691419

[B100] Sorokin D. Y. Tourova T. P. Mußmann M. Muyzer G. (2008). *Dethiobacter alkaliphilus* gen. nov. sp. nov., and *Desulfurivibrio alkaliphilus* gen. nov. sp. nov.: Two novel representatives of reductive sulfur cycle from soda lakes. *Extremophiles* 12 431–439. 10.1007/s00792-008-0148-8 18317684

[B101] Staley C. Gould T. J. Wang P. Phillips J. Cotner J. B. Sadowsky M. J. (2016). Sediments and soils act as reservoirs for taxonomic and functional bacterial diversity in the upper Mississippi River. *Microb. Ecol.* 71 814–824. 10.1007/s00248-016-0729-5 26879939

[B102] Stauffert M. Duran R. Gassie C. (2014). Response of archaeal communities to oil spill in bioturbated mudflat sediments. *Microb. Ecol.* 67 108–119. 24057322 10.1007/s00248-013-0288-y

[B103] Sugihara C. Yanagawa K. Okumura T. Takashima C. Harijoko A. Kano A. (2016). Transition of microbiological and sedimentological features associated with the geochemical gradient in a travertine mound in northern Sumatra, Indonesia. *Sediment. Geol.* 343 85–98. 10.1016/j.sedgeo.2016.07.012

[B104] Terrisse F. Cravo-Laureau C. Noël C. Cagnon C. Dumbrell A. J. McGenity T. J. (2017). Variation of oxygenation conditions on a hydrocarbonoclastic microbial community reveals *Alcanivorax* and *Cycloclasticus* ecotypes. *Front. Microbiol.* 8:1549. 10.3389/fmicb.2017.01549 28861063 PMC5562018

[B105] Tu D. Ke J. Luo Y. Hong T. Sun S. Han J. (2022). Microbial community structure and shift pattern of industry brine after a long-term static storage in closed tank. *Front. Microbiol.* 13:975271. 10.3389/fmicb.2022.975271 36118215 PMC9478951

[B106] Ugalde J. A. Narasingarao P. Kuo S. Podell S. Allen E. E. (2013). Draft genome sequence of “*Candidatus Halobonum tyrrellensis*” strain G22, isolated from the hypersaline waters of Lake Tyrrell, Australia. *Genome Announc.* 1:e01001-13. 10.1128/genomeA.01001-13 24336364 PMC3861417

[B107] Wang H. Bier R. Zgleszewski L. Peipoch M. Omondi E. Mukherjee A. (2020). Distinct distribution of archaea from soil to freshwater to estuary: Implications of archaeal composition and function in different environments. *Front. Microbiol.* 11:576661. 10.3389/fmicb.2020.576661 33193193 PMC7642518

[B108] Wang Y. Qian P.-Y. (2009). Conservative fragments in bacterial 16S RRNA genes and primer design for 16s ribosomal DNA amplicons in metagenomic studies. *PLoS One* 4:7401. 10.1371/journal.pone.0007401 19816594 PMC2754607

[B109] Wecker P. Klockow C. Ellrott A. Quast C. Langhammer P. Harder J. (2009). Transcriptional response of the model planctomycete *Rhodopirellula baltica* SH1Tto changing environmental conditions. *BMC Genomics* 10:410. 10.1186/1471-2164-10-410 19725962 PMC2754498

[B110] Williams W. D. (1996). The largest, highest and lowest lakes of the world: Saline lakes. *Verh. Int. Verein Limnol.* 26 61–79. 10.1080/03680770.1995.11900693

[B111] Williams W. D. (2002). Environmental threats to salt lakes and the likely status of inland saline ecosystems in 2025. *Environ. Conserv.* 29 154–167. 10.1017/S0376892902000103

[B112] Wu S. Wang J. Wang J. Du X. Ran Q. Chen Q. (2022). *Halalkalibacterium roseum* gen. nov., sp. nov., a new member of the family Balneolaceae isolated from soil. *Int. J. Syst. Evol. Microbiol.* 72:005339. 10.1099/ijsem.0.005339 35482520

[B113] Youssef N. H. Ashlock-Savage K. N. Elshahed M. S. (2012). Phylogenetic diversities and community structure of members of the extremely halophilic archaea (order halobacteriales) in multiple saline sediment habitats. *Appl. Environ. Microbiol.* 78 1332–1344. 10.1128/AEM.07420-11 22179255 PMC3294467

[B114] Yuan C. Na S. Li F. Hu H. (2021). Impact of sulfate and iron oxide on bacterial community dynamics in paddy soil under alternate watering conditions. *J. Hazard. Mater.* 408:124417. 10.1016/j.jhazmat.2020.124417 33172683

[B115] Yue Y. Shao T. Long X. He T. Gao X. Zhou Z. (2020). Microbiome structure and function in rhizosphere of Jerusalem artichoke grown in saline land. *Sci. Total Environ.* 724:138259. 10.1016/j.scitotenv.2020.138259 32247981

[B116] Zeng Y. Selyanin V. Lukeŝ M. Dean J. Kaftan D. Feng F. (2015). Characterization of the microaerophilic, bacteriochlorophyll a-containing bacterium *Gemmatimonas phototrophica* sp. Nov., and emended descriptions of the genus *Gemmatimonas* and *Gemmatimonas aurantiaca*. *Int. J. Syst. Evol. Microbiol.* 65 2410–2419. 10.1099/ijs.0.000272 25899503

[B117] Zhang X. Y. Zhang R. Wang J. C. Zhang T. Du Z. J. (2020). *Wenzhouxiangella limi* sp. Nov., isolated from a salt lake. *Int. J. Syst. Evol. Microbiol.* 70 4610–4615. 10.1099/ijsem.0.004320 32658636

[B118] Zhu J. He Y. Zhu Y. Huang M. Zhang Y. (2018). Biogeochemical sulfur cycling coupling with dissimilatory nitrate reduction processes in freshwater sediments. *Environ. Rev.* 26 121–132. 10.1139/er-2017-0047

