# Supplementary material for: Vertical organization of microbial communities in Salineta hypersaline wetland, Spain
Source: Front Microbiol. 2023 Jan 27;14:869907. doi: 10.3389/fmicb.2023.869907 (PMC9911865; doi:10.3389/fmicb.2023.869907)
Supplement: Supplementary file 1 [file Data_Sheet_1.docx]

## **Supplementary Materials**

## **Supplementary Information**

**Supplementary Figure S1.** Transect layers (cores) from S1, S2 and S3 stations corresponding respectively to usually submerged soil, intermittently flooded soil and soil covered with halophytes. The cores were sampled in winter showing the layers (L1, L2, L3 for S1; L1, L2 for S2 and S3).

**Supplementary Figure S2.** Comparison of physical-chemical parameters. Non-metric multidimensional scale (NMDS) in the different layers (L1, L2, L3 or L1, L2) from each station (S1, S2, S3) at winter (wet period) and summer (dry period). The significant environmental variables are represented by vector.

**Supplementary Figure S3.** Rarefaction curves based on 16S rRNA gene MiSeq Illumina sequencing. Winter, W (wet period); Summer, S (dry period); Stations: S1, S2, S3; Layers: L1, L2, L3; Replicate: R1, R2, R3. The curves were calculated with singletons using the "rarecurve" function in Vegan package.

**Supplementary Table S1.** Gaz flux production from the study site located at stations S1, S2, and S3 in Salineta wetland in winter (W, wet period) and summer (S, dry period). Mean ± SD are presented (n = 4). The same small letter indicates no significant difference of means compared by ANOVA, *p* < 0.05.

**Supplementary Table S2.** Physical-chemical parameters in the different layers (L1, L2, L3 or L1, L2) from each station (S1, S2, S3) at winter (W, wet period) and summer (S, dry period) at Salineta wetland. Mean ± SD are presented (n = 3, except for S.S1.L2, n = 2). The same small letter indicates no significant difference of means compared by ANOVA, *p* < 0.05.

**
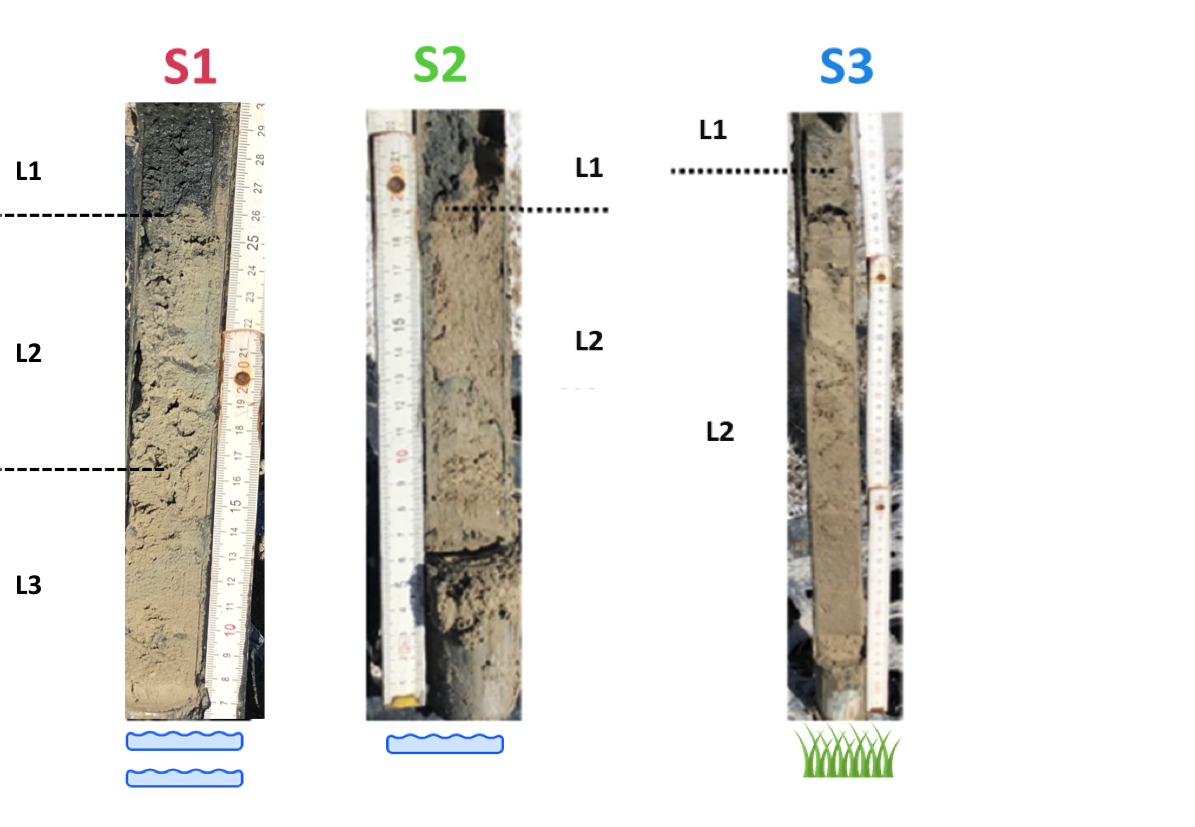
**

**Supplementary Figure S1.** Transect layers (cores) from S1, S2 and S3 stations corresponding respectively to usually submerged soil, intermittently flooded soil and soil covered with halophytes. The cores were sampled in winter showing the layers (L1, L2, L3 for S1; L1, L2 for S2 and S3).


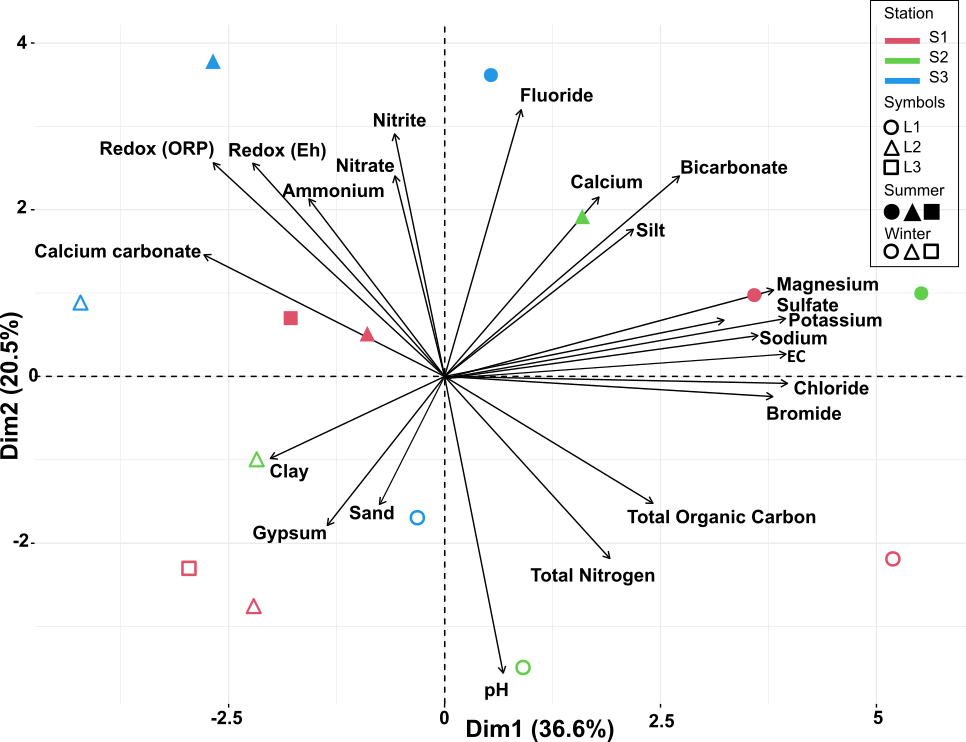


**Supplementary Figure S2.** Comparison of physical-chemical parameters. Non-metric multidimensional scale (NMDS) in the different layers (L1, L2, L3 or L1, L2) from each station (S1, S2, S3) at winter (wet period) and summer (dry period). The significant environmental variables are represented by vector.


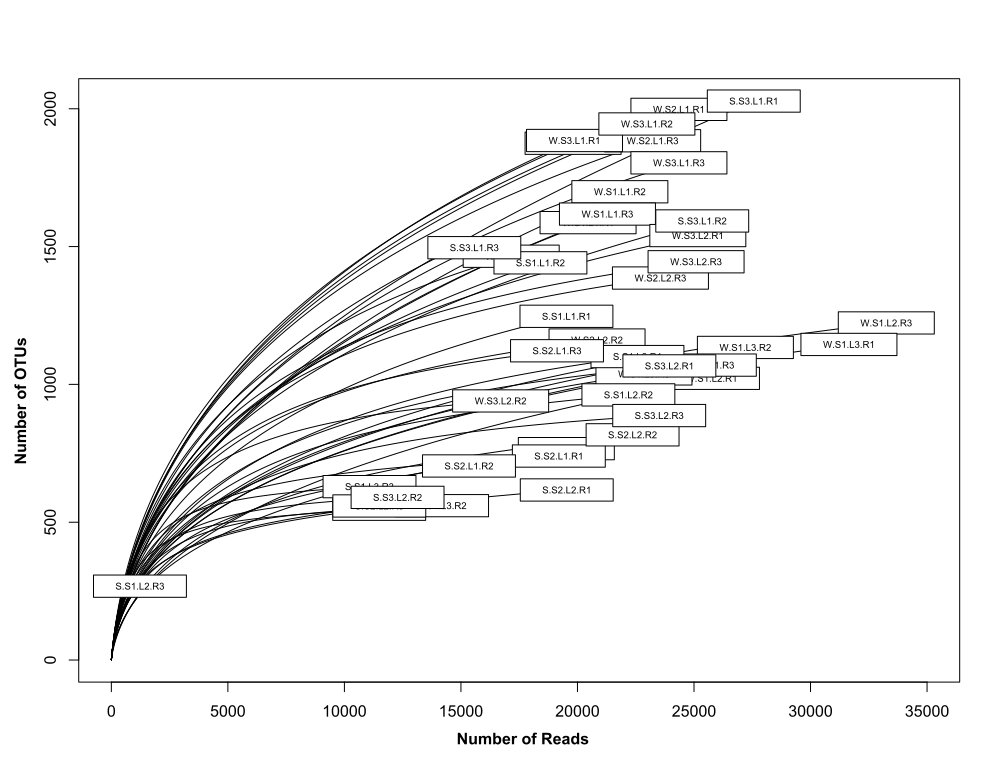


**Supplementary Figure S3.** Rarefaction curves based on 16S rRNA gene MiSeq Illumina sequencing. Winter, W (wet period); Summer, S (dry period); Stations: S1, S2, S3; Layers: L1, L2, L3; Replicate: R1, R2, R3.

**Supplementary Table S1.** Gaz flux production from the study site located at stations S1, S2, and S3 in Salineta wetland in winter (W, wet period) and summer (S, dry period). Mean ± SD are presented (n = 4). The same small letter indicates no significant difference of means compared by ANOVA, *p* < 0.05.

| Gaz  Flux (mgm^-2^day^-1^) |  | |  | | Stations |  |
| --- | --- | --- | --- | --- | --- | --- |
|  | **Seasons** | **S1** | | **S2** | | **S3** |
| CO_2_ | W | 30.21 ± 93.4^b^ | | -13.73 ± 81.4^b^ | | 425.63 ± 210.2^a^ |
|  | S | 63.02 ± 82.5^b^ | | 37.07 ± 54.8^b^ | | 318.80 ± 129.6^a^ |
| CH_4_ | W | 0 ± 0.15^b^ | | 0.10 ± 0.2^ab^ | | 0.73 ± 0.5^a^ |
|  | S | -0.05 ± 0.13^a^ | | -0.14 ± 0.04^a^ | | -0.33 ± 0.2^a^ |
| N_2_O | W | -0.094 ± 0.1^a^ | | -0.019 ± 0.2^a^ | | -0.125 ± 0.2^a^ |
|  | S | 0.588 ± 0.35^a^ | | 0.520 ± 0.5^a^ | | -0.049 ± 0.17^a^ |

**Supplementary Table S2.** Physical-chemical parameters in the different layers (L1, L2, L3 or L1, L2) from each station (S1, S2, S3) at winter (W, wet period) and summer (S, dry period) at Salineta wetland. Mean ± SD are presented (n = 3, except for S.S1.L2, n = 2). The same small letter indicates no significant difference of means compared by ANOVA, *p* < 0.05.

| **Physical-chemical parameter** | | **Stations** | | | | | | |
| --- | --- | --- | --- | --- | --- | --- | --- | --- |
|  | | **S1** | | | **S2** | | **S3** | |
| **Compounds** | | L1 | L2 | L3 | L1 | L2 | L1 | L2 |
| **Redox (ORP, mV)** | W  S | -334 ± 4^g^-130 ± 4.4^f^ | -76 ± 4^d^-53 ± 1^e^ | 65 ± 5^c^0^d^ | -254 ± 14^e^-155 ± 20^g^ | 191 ± 14.7^a^107 ± 9^c^ | -291 ± 9^f^234 ± 7.2^a^ | 137 ± 9^b^224 ± 10.5^b^ |
| **Redox (Eh. mV)** | W  S | -134 ± 3^g^  70 ± 2^e^ | 124 ± 1^d^  147 ± 1^d^ | 265 ± 1.5^c^  0^g^ | -54 ± 2^e^  45 ± 2^f^ | 391 ± 1.5^a^  307 ± 3.4^c^ | -91 ± 2^f^  434 ± 1.5^a^ | 337 ± 2^b^  424 ± 3^b^ |
| **pH** | W  S | 8.9 ± 0.06^ab^  8.6 ± 0.17^a^ | 8.9 ± 0.01^ab^  8.22 ± 0.06^abc^ | 8.7 ± 0.06^bc^  8.1 ± 0.11^abc^ | 9.1 ± 0.14^a^  8.4 ± 0.15^ab^ | 8.7 ± 024^bc^  8 ± 0.13b^c^ | 8.8 ± 0.03^bc^  8.4 ± 0.14^ab^ | 8.5 ± 0.02^c^  7.8 ± 0.30^c^ |
| **EC (dS/m 1:5v)** | W  S | 35.8 ± 5.02^a^  50.3 ± 5.42^a^ | 14.2 ± 2.40^bc^  21.03 ± 2.05^c^ | 12.2 ± 1.41^bc^  17.3 ± 1.05^b^ | 27.2 ± 14.65^ab^  55.5 ± 16.15^a^ | 18.5 ± 1.07^abc^  27.7 ± 6.28^b^ | 14.3 ± 3.12^bc^  25.1 ± 4.78^b^ | 6.4 ± 1.47^c^  12.3 ± 1.4^b^ |
| **Calcium carbonate equivalent (%)** | W  S | 14.9 ± 1.73^c^  7.3 ± 2.52^d^ | 18.2 ± 0.45^bc^  15.5 ± 1.9^bcd^ | 20.4 ± 0.97^bc^  17.3 ± 1.43^bc^ | 13.4 ± 3.91^c^  9.8 ± 2.46^cd^ | 17.9 ± 1.34^bc^  17.8 ± 1.63^bc^ | 29.8 ± 7.31^ab^  23.9 ± 5.77^b^ | 34.9 ± 6.45^a^  39.6 ± 4.47^a^ |
| **Gypsum (%)** | W  S | 28.1 ± 1.04^b^  25.7 ± 3.97^ab^ | 40.8 ± 2.55^ab^  43.55 ± 4.7^a^ | 35.1 ± 0.39^b^  40.9 ± 4.84^a^ | 50.9 ± 3.11^a^  25.1 ± 11.36^ab^ | 50.7 ± 3.37^a^  37.5 ± 6.68^ab^ | 27.9 ± 8.09^b^  36.7 ± 4.57^ab^ | 33.3 ± 7.56^b^  20.2 ± 3.19^b^ |
| **Total Organic Carbon (Dicromate) (%)** | W  S | 1.6 ± 0.02^a^  0.6 ± 0.01^a^ | 0.5 ± 0.03^c^  0.54 ± 0.03^a^ | 0.4 ± 0.004^c^  0.3 ± 0.02^a^ | 0.9 ± 0.12^b^  0.5 ± 0.46^a^ | 0.5 ± 0.07^c^  0.6 ± 0.09^a^ | 1 ± 0.01^b^  0.7 ± 0.07^a^ | 0.4 ± 0.04^c^  0.5 ± 0.06^a^ |
| **Total Organic Carbon (LECO) (%)** | W  S | 2.7 ± 0.29^a^  1 ± 0.48^a^ | 1 ± 0.18b^c^  1.2 ± 0.22^a^ | 1.1 ± 0.25^bc^  1 ± 0.37^a^ | 0.7 ± 0.34^c^  0.6 ± 0.12^a^ | 0.7 ± 0.40^c^  1.1 ± 0.19^a^ | 1.7 ± 0.42^b^  1.2 ± 0.18^a^ | 0.3 ± 0.06^c^  1.2 ± 0.26^a^ |
| **Total Nitrogen (%)** | W  S | 0.2 ± 0.02^a^  0.04 ± 0.01^ab^ | 0.1 ± 0.001^cd^  0.03 ± 0.023^ab^ | 0.1 ± 0.02^cd^  0.02 ± 0.01^b^ | 0.1 ± 0.01^bc^  0.1 ± 0.02^ab^ | 0.04 ± 0.01^cd^  0.05 ± 0.01^ab^ | 0.1 ± 0.01^b^  0.1 ± 0.01^a^ | 0.04 ± 0.01^d^  0.04 ± 0.002^ab^ |
| **Clay (%)** | W  S | 36.3 ± 0.96^cd^  34.5 ± 10.15^ab^ | 48.9 ± 0.32^a^  31.35 ± 8.56^ab^ | 46.9 ± 1.93^ab^  48.5 ± 8.84^a^ | 28.1 ± 5.92^d^  30.1 ± 3.02^ab^ | 39.3 ± 2.84^bc^  35.9 ± 4.71^ab^ | 29.2 ± 3.29^d^  19.4 ± 5.94^b^ | 35.6 ± 0.10^cd^  42.6 ± 2.62^ab^ |
| **Silt (%)** | W  S | 51.4 ± 1.06^a^  38.6 ± 6.54^bc^ | 24 ± 6.13^c^  22.84 ± 3.54^cd^ | 20.8 ± 1.09^c^  21.8 ± 3.65^d^ | 24.4 ± 8.69^c^  42.4 ± 6.09^ab^ | 28.2 ± 2.25^c^  42 ± 9.58^ab^ | 49.8 ± 1.64^ab^  56 ± 3.19^a^ | 36 ± 7.45^bc^  39.5 ± 2.23^b^ |
| **Sand (%)** | W  S | 12.3 ± 0.10^b^  26.9 ± 11.66^a^ | 27.1 ± 5.81^ab^  45.8 ± 5.01^a^ | 32.3 ± 3.02^ab^  29.6 ± 12.47^a^ | 47.5 ± 14.61^a^  27.4 ± 8.17^a^ | 32.6 ± 2.95^ab^  22 ± 4.92^a^ | 21 ± 1.65^b^  24.6 ± 3.31^a^ | 28.4 ± 7.54^ab^  17.8 ± 0.42^a^ |
| **Fluoride (mg/L)** | W  S | 0.009 ± 0.02^a^  0.2 ± 0.03^a^ | 0.035 ± 0.01^a^  0.11± 0.004^a^ | 0.023 ± 0.004^a^  0.1 ± 0.06^a^ | 0.038 ± 0^a^  0.1 ± 0.03^a^ | 0.029 ± 0.01^a^  0.1 ± 0.02^a^ | 0.009 ± 0.02^a^  0.1 ± 0.01^a^ | 0.07 ± 0.08^a^  0.1 ± 0.01^a^ |
| **Chloride (mg/L)** | W  S | 8185.3 ± 103.25^a^  5831.3 ± 405.7^abc^ | 3003.8 ± 1114.4^bc^  4684.4 ± 535.07^abc^ | 2614.4 ± 69.79^bc^  3454 ± 239.79^bc^ | 5834.7 ± 3267.95^ab^  8085.4 ± 2581.97^a^ | 4156.8 ± 164.3^bc^  6592.3 ± 1096.4^ab^ | 2806.8 ± 536.75^bc^  4899.4 ± 636.57^abc^ | 869.1 ± 308.69^c^  2672.7 ± 447.64^c^ |
| **Bromide (mg/L)** | W  S | 6.9 ± 0.13^a^  4.4 ± 0.31^abc^ | 2.6 ± 1.03^bc^  4.1 ± 0.5^abc^ | 2.2 ± 0.005^bc^  2.9 ± 0.31^c^ | 5.1 ± 2.9^ab^  6.3 ± 1.39^a^ | 3.6 ± 0.22^abc^  5.9 ± 1.16^ab^ | 2.4 ± 0.39^bc^  3.8 ± 0.19^bc^ | 0.7 ± 0.22^c^  2.4 ± 0.44^c^ |
| **Nitrite (mg/L)** | W  S | 0^a^  0^a^ | 0^a^  0^a^ | 0^a^  0^a^ | 0^a^  0^a^ | 0^a^  0^a^ | 0^a^  0.8 ± 0.42^a^ | 0.009 ± 0.02^a^  0.7 ± 1.15^a^ |
| **Nitrate (mg/L)** | W  S | 0^a^  0.2 ± 0.42^b^ | 0.8 ± 0.24^a^  0.44 ± 0.17^b^ | 0.1 ± 0.02^a^  0.5 ± 0.43^b^ | 0.1 ± 0.06^a^  0^b^ | 0.6 ± 0.55^a^  0.1 ± 0.21^b^ | 0^a^  6.6 ± 1.68^a^ | 1 ± 1.05^a^  1.8 ± 0.67^b^ |
| **Sulfate (mg/L)** | W  S | 9826.5 ± 2119.24^a^  33703.2 ± 5124.2^a^ | 5291.3 ± 545.35^a^  6580.4 ± 149.6^b^ | 3790.4 ± 825.1^a^  5418.5 ± 32.57^b^ | 10194.4 ± 6124.4^a^  33855.7 ± 13911^a^ | 5160.9 ± 704.52^a^  7871.5 ± 2283.4^b^ | 4053.5 ± 1612.72^a^  9979.5 ± 2302.1^b^ | 2720 ± 87.57^a^  3633 ± 86.65^b^ |
| **Bicarbonate (mg/L)** | W  S | 223.7 ± 88.07^a^  223.7 ± 127.02^a^ | 81.4 ± 35.23^a^  213.6 ± 43.15^a^ | 122 ± 0^a^  223.7 ± 35.23^a^ | 132.2 ± 35.23^a^  325.4 ± 35.23^a^ | 91.5 ± 0^a^  244.1 ± 183.06^a^ | 142.4 ± 88.07^a^  183.1 ± 105.7^a^ | 122 ± 52.84^a^  244.1 ± 105.7^a^ |
| **Sodium (mg/L)** | W  S | 8015.2 ± 1756.94^a^  17201.5 ± 3233^ab^ | 3506.4 ± 753.67^abc^  4273.6 ± 446.4^c^ | 2532.8 ± 294.64^bc^  3618.2 ± 467.44^c^ | 6383.8 ± 3802.7^ab^  19614 ± 7570.03^a^ | 3309.8 ± 176.63^abc^  6835.5 ± 3074.36^bc^ | 2397.2 ± 598.95^bc^  5740.8 ± 2250.5^c^ | 827 ± 246.67^c^  2019.8 ± 365.04^c^ |
| **Ammonium (mg/L)** | W  S | 0^a^  0^b^ | 0^a^  2.38 ± 0.03^ab^ | 0^a^  2.9 ± 0.12^a^ | 0^a^  0^b^ | 0.2 ± 0.4^a^  0.5 ± 0.86^b^ | 0.5 ± 0.44^a^  1.4 ± 1.38^ab^ | 1 ± 0.97^a^  1.4 ± 0.46^ab^ |
| **Potassium (mg/L)** | W  S | 314 ± 25.16^a^  241 ± 14.55^ab^ | 124.9 ± 44.45^b^  193.5 ± 18.5^ab^ | 101.7 ± 3.37^b^  158.8 ± 18.63^ab^ | 194.1 ± 100.14^ab^  296 ± 67.92^a^ | 137.9 ± 15.69^b^  296.2 ± 88.63^a^ | 137.5 ± 17.91^b^  231.7 ± 59.75^ab^ | 69.1 ± 0.8^b^  135.5 ± 22.86^b^ |
| **Calcium (mg/L)** | W  S | 800.9 ± 8.44^a^  698.5 ± 13.9^a^ | 428 ± 181.43^bc^  728 ± 5.5^a^ | 306 ± 55.91^c^  764.5 ± 53.7^a^ | 612.4 ± 168.55^abc^  711.1 ± 2.95^a^ | 739.9 ± 86.1^ab^  864.7 ± 118.32^a^ | 732.2 ± 100.73^ab^  727.7 ± 96.27^a^ | 660 ± 45.72^ab^  727.6 ± 55.96^a^ |
| **Magnesium (mg/L** | W  S | 1651.5 ± 242.21^a^  1680.1 ± 149.22^ab^ | 493.3 ± 180.06^bc^  856.3 ± 119^ab^ | 455.7 ± 2.29^bc^  813.4 ± 123.15^b^ | 1106.7 ± 620.53^ab^  2337.9 ± 777.51^a^ | 820.4 ± 102.06^abc^  1452.1 ± 468.49^ab^ | 591.1 ± 204^bc^  1756.6 ± 639.81^ab^ | 178.4 ± 44.97462.2 462.2 ± 81.80^b^ |
